# Nonconserved epitopes dominate reverse preexisting T cell immunity in COVID-19 convalescents

**DOI:** 10.1038/s41392-024-01876-3

**Published:** 2024-06-12

**Authors:** Xin Wang, Jie Zhang, Maoshun Liu, Yuanyuan Guo, Peipei Guo, Xiaonan Yang, Bingli Shang, Min Li, Jinmin Tian, Ting Zhang, Xi Wang, Ronghua Jin, Jikun Zhou, George F. Gao, Jun Liu

**Affiliations:** 1 Department of Epidemiology, School of Public Health, Cheeloo College of Medicine, Shandong University, Jinan, 250012 China; 2 NHC Key Laboratory of Biosafety, Research Unit of Adaptive Evolution and Control of Emerging Viruses, Chinese Academy of Medical Sciences, National Institute for Viral Disease Control and Prevention, Chinese Center for Disease Control and Prevention (China CDC), Beijing, 102206 China; 3 Beijing Key Laboratory of Emerging Infectious Diseases, Institute of Infectious Diseases, Beijing Ditan Hospital, Capital Medical University, Beijing, 100015 China; 4 Beijing Institute of Infectious Diseases, Beijing, 100015 China; 5 National Center for Infectious Diseases, Beijing Ditan Hospital, Capital Medical University, Beijing, 100015 P.R. China; 6 School of Laboratory Medicine and Life Sciences, Wenzhou Medical University, Wenzhou, 325035 China; 7 School of Ophthalmology & Optometry, Wenzhou Medical University, Wenzhou, 325027 China; 8 Collaborative Innovation Centre of Regenerative Medicine and Medical BioResource Development and Application Co-constructed by the Province and Ministry, Guangxi Medical University, Nanning, Guangxi 530021 China; 9 Shijiazhuang Fifth Hospital, Shijiazhuang, 050011 China; 10 CAS Key Laboratory of Pathogen Microbiology and Immunology, Institute of Microbiology, Chinese Academy of Sciences (CAS), Beijing, 100101 China; 11 Savaid Medical School, University of Chinese Academy of Sciences, Beijing, 100049 China

**Keywords:** Infectious diseases, Antigen processing and presentation

## Abstract

The herd immunity against SARS-CoV-2 is continuously consolidated across the world during the ongoing pandemic. However, the potential function of the nonconserved epitopes in the reverse preexisting cross-reactivity induced by SARS-CoV-2 to other human coronaviruses is not well explored. In our research, we assessed T cell responses to both conserved and nonconserved peptides shared by SARS-CoV-2 and SARS-CoV, identifying cross-reactive CD8^+^ T cell epitopes using enzyme-linked immunospot and intracellular cytokine staining assays. Then, in vitro refolding and circular dichroism were performed to evaluate the thermal stability of the HLA/peptide complexes. Lastly, single-cell T cell receptor reservoir was analyzed based on tetramer staining. Here, we discovered that cross-reactive T cells targeting SARS-CoV were present in individuals who had recovered from COVID-19, and identified SARS-CoV-2 CD8^+^ T cell epitopes spanning the major structural antigens. T cell responses induced by the nonconserved peptides between SARS-CoV-2 and SARS-CoV were higher and played a dominant role in the cross-reactivity in COVID-19 convalescents. Cross-T cell reactivity was also observed within the identified series of CD8^+^ T cell epitopes. For representative immunodominant peptide pairs, although the HLA binding capacities for peptides from SARS-CoV-2 and SARS-CoV were similar, the TCR repertoires recognizing these peptides were distinct. Our results could provide beneficial information for the development of peptide-based universal vaccines against coronaviruses.

## Introduction

The COVID-19 pandemic, caused by SARS-CoV-2 infection, has led to over 600 million cases and more than 6 million deaths globally (https://covid19.who.int/), which brings serious challenges to the public health. SARS-CoV-2 is widely susceptible and highly contagious, spreading rapidly worldwide. Following SARS-CoV-2 infection, clinical manifestations can range from asymptomatic or mild respiratory symptoms to severe respiratory diseases or death.^1,2^. The adaptive immune response of the body is crucial in dealing with SARS-CoV-2 infection. But in fact, SARS-CoV-2 circulation across the world has changed the background of the baseline immunity against coronaviruses among the population, which may influence the clinical manifestations of human coronaviruses, including the reinfection of SARS-CoV-2.

Many studies have found that the titers of specific antibodies in infected individuals decreased over time.^3,4^ Some asymptomatic and mild infected individuals may not produce protective humoral immune response after infection, but strong T cell immune response.^5^ To provide information for therapeutic interventions and vaccine design, it is essential to understand how T cell immunity affects the pathogenesis of COVID-19 and long-term protective immunity.^6^ SARS-CoV-2 antigen-specific T cell responses have been identified and seem to last longer than specific antibodies.^7^ SARS-CoV-2-specific cellular immune responses can be detected at least until one year after infection.^8^ Additionally, these T cells can be activated by SARS-CoV-2 without seroconversion, indicating that T cell immunity can safeguard individuals with congenital or acquired deficiencies in humoral immunity. The multiple SARS-CoV-2 variants can escape the neutralization of humoral immunity induced by vaccines and attenuate the effectiveness of vaccines,^9,10^ but more than 80% of vaccine induced memory T cell responses against SARS-CoV-2 variants can be preserved.^11,12^ In addition, infected individuals with strong N-specific T cell responses also had a slower decrease in N-specific antibodies after half a year, indicating that T cell immunity may support the production of antibodies.^13^ To sum up, high levels of neutralizing antibodies can prevent and control SARS-CoV-2 infection, but preventing reinfection and avoiding severe diseases mainly rely on T cells when the antibodies are insufficient.

SARS-CoV-2, belongs to the *Coronaviridae* family, is the seventh human coronavirus (HCoV). Among human coronaviruses, HCoV-OC43, HCoV-HKU1, HCoV-229E, and HCoV-NL63 are endemic and can cause the common cold.^14^ Severe acute respiratory syndrome coronavirus (SARS-CoV), Middle East respiratory syndrome coronavirus (MERS-CoV), and SARS-CoV-2 are also the human-infecting coronaviruses and can cause severe pneumonia.^15^ Among them, MERS-CoV belongs to the *Merbecovirus* subgenus, while SARS-CoV and SARS-CoV-2 both belong to *Sarbecovirus* subgenus. T cell immunity can exert long-term protection and has great potential to generate cross-reactivity within these coronaviruses.^16^ In spite of high antigen-specificity, T cell immunity can easily cross-recognize highly conserved epitopes.^17^ The sequence of SARS-CoV-2 has about 80% homology to the sequence of SARS-CoV.^18^ The homology of their main structural proteins is 75.7% for spike protein (S), 90.5% for membrane protein (M) and 90.5% for nucleocapsid protein (N), respectively. Post-infection T cell immunity from SARS-CoV-2 has significant potential for cross-reactivity with these viruses due to its prolonged duration. Cellular immunity against immunodominant CD8^+^ T cell epitopes can provide protection against lethal viral infections.^19^ At present, there is no effective therapeutic drugs for seriously harmful HCoVs, and high vigilance should be maintained in preventing and controlling their infection Therefore, it is important to find relatively conserved T cell epitopes for exploring cross-immunity between viruses.

Currently, most studies on the cross-reactivity between SARS-CoV-2 and SARS-CoV mainly focused on the cross-recognition of neutralizing antibodies. Some SARS-CoV RBD-specific neutralizing antibodies (nAbs) are reported to have cross-neutralization activity against SARS-CoV-2, as they target highly conserved sites shared by these two viruses.^20–28^ Conversely, the nAbs from SARS-CoV-2-specific B cell repertoire can also potently neutralized SARS-CoV and the mutants.^29–31^ While respiratory virus infections may induce transient antibody responses, virus-specific T cells generally exhibit greater persistence. SARS-CoV-specific memory T cells have been detected even 17 years after SARS and it indicated that the previously infected SARS patients could have preexisting cellular immunity to SARS-CoV-2.^32^ And the T cells showed strong cross-reactivity to the N protein of SARS-CoV-2, which has more cross-reactive epitopes and is relatively conserved with the sequence of SARS-CoV. A study showed that infected mice exhibited cross-reactive T cell responses between SARS-CoV and SARS-CoV-2.^33^ And at present, the cross-reactive CD8^+^ T cell epitopes between SARS-CoV and SARS-CoV-2 had only been described in mice. However, the reverse preexisting immunity and cross-T cell barrier established by SARS-CoV-2 to SARS-CoV, and whether the nonconserved epitopes matters within this process are still largely unknown. In this study, we examined the presence of cross-reactive SARS-CoV-specific T cells in COVID-19 convalescents and assessed T cell responses to both conserved and nonconserved peptides of SARS-CoV-2 and SARS-CoV.We also identified the cross-reactive CD8^+^ T cell epitopes of SARS-CoV-2 and SARS-CoV. Our findings may offer valuable insights into the immune escape mechanisms of human-infecting coronaviruses and the design of vaccines against SARS-CoV-2 and other coronaviruses.

## Results

### SARS-CoV-2-specific T cells in COVID-19 convalescents are cross-reactive to SARS-CoV

**Fig. 1:**
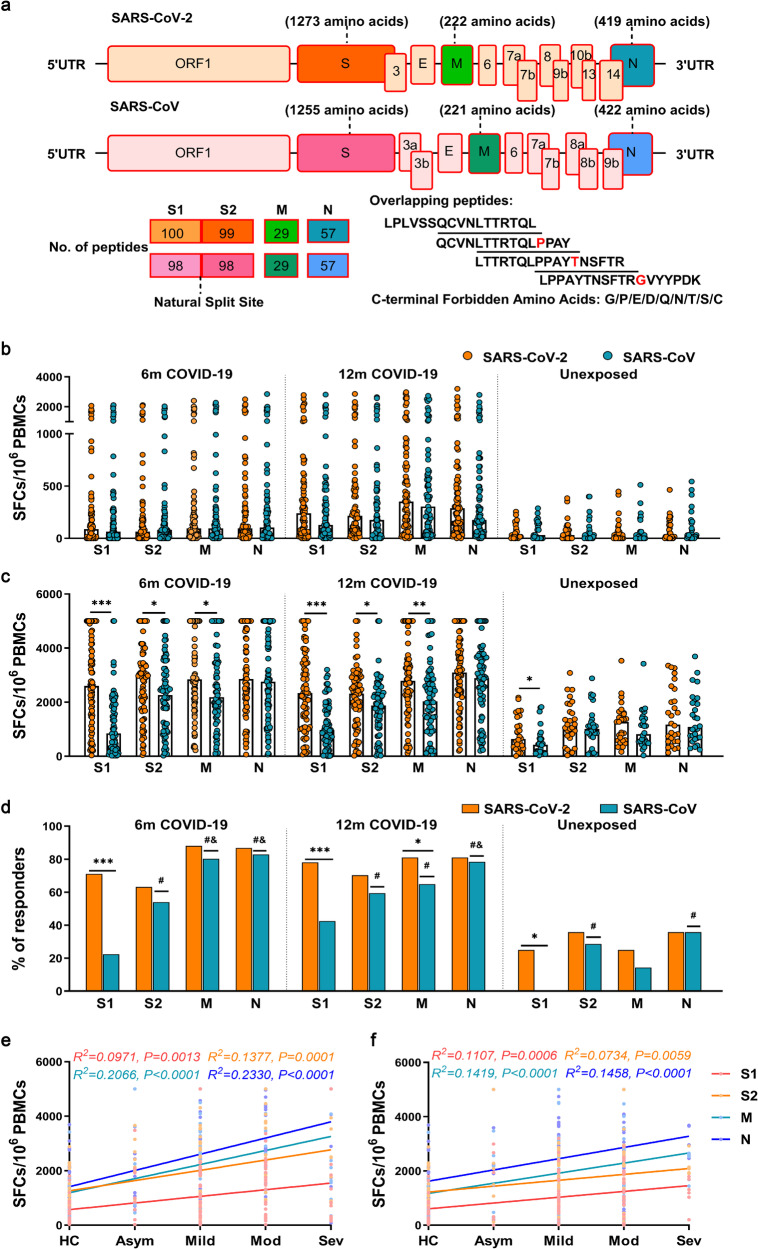
Cross-reactive T cell responses to SARS-CoV in COVID-19 convalescents. **a** The proteome organization of SARS-CoV-2 and SARS-CoV. The 15- to 18-mer peptides overlapped by 10 amino acids spanning the S, M and N proteins were split into 4 peptide pools according to the natural split sites. PBMCs of COVID-19 convalescents at 6 months (6 m, left; *n* = 76) and 12 months (12 m, middle; *n* = 74) after disease onset and PBMCs of healthy controls (HCs, right; *n* = 28) were stimulated with the 4 peptide pools of SARS-CoV-2 and SARS-CoV freshly ex vivo (**b**) or after an expansion in vitro (**c**). **d** The percentages of COVID-19 convalescents at 6 m and 12 m and HCs with positive T cell responses to different peptide pools of SARS-CoV-2 and SARS-CoV. The “#” or “&” symbols indicate significant differences comparing with S1 or S2 peptide pool, respectively. The effect of disease severity (Healthy controls (HCs), Asymptomatic (Asym), Mild (Mild), Moderate (Mod), and Severe (Sev)) on the cross-recognition levels of SARS-CoV in COVID-19 convalescents at 6 m (**e**) and 12 m (**f**). R^2^ represents the goodness of fit. Data are presented as median or N (%). A Mann–Whitney U-test was used in **b** and **c**. A chi-square test or Fisher’s exact test was used in **d**. A simple linear regression was used in **e** and **f**. Two-tailed *P* values were calculated. * *P* < 0.05; ** *P* < 0.01; *** *P* < 0.001

We collected peripheral blood samples from 95 COVID-19 convalescents at 6 months (6 m, *n* = 76) and 12 months (12 m, *n* = 74) after disease onset and detected the T cell responses against SARS-CoV-2 and SARS-CoV by using the IFN-γ ELISpot assay. First, freshly isolated PBMCs (ex vivo) were stimulated with the 4 pools of overlapping peptides covering the S (including S1 and S2), M and N proteins (Fig. 1a) and then tested with ELISpot assay, which showed that the T cell responses to SARS-CoV-2 and SARS-CoV were weak and had no significant difference (*P* > 0.05) (Fig. 1b).

Next, PBMCs were expanded for 9 days with 4 peptide pools of SARS-CoV-2. After expansion, the T cell responses to S1, S2 and M peptide pools of SARS-CoV-2 were significantly higher than those of SARS-CoV both at 6 m and 12 m (*P* < 0.05). However, there was no significant difference in the T cell responses to N protein (*P* > 0.05) (Fig. 1c). Besides, 22%-42% COVID-19 convalescents had responses to S1 of SARS-CoV, whereas 78%-83% had responses to the N protein (Fig. 1d). At 6 months, 89% (68/76) of COVID-19 convalescents had positive T cell responses to at least one SARS-CoV peptide pool, while decreasing to 82% (61/74) at 12 months. Our results showed that COVID-19 convalescents had strong cross-reactivity to SARS-CoV and the N of SARS-CoV could induce stronger cross-reaction than other structure proteins. We observed positive T cell responses to both SARS-CoV-2 and SARS-CoV in healthy controls (HCs), likely indicating cross-reactivity with common cold coronaviruses prevalent in the healthy population. No significant difference was observed between peptide pools of these two viruses, except S1 after in vitro expansion (Fig. 1b–d).

Furthermore, we also analyzed the relationship between the cross-recognition levels of SARS-CoV in COVID-19 convalescents and different disease severities. We transformed the disease severity into a rank variable and performed a simple linear regression analysis.The HCs were considered as the lowest rank in the regression analysis. There was a relatively good fit between disease severity and the T cell responses to different peptide pools of SARS-CoV both at 6 m and 12 m, showing an rising trend for cross-recognition levels in COVID-19 convalescents with increasing disease severity (Fig. 1e, f).

### Identification of positive SARS-CoV-2 overlapping peptides and CD8^+^ T cell epitopes

**Fig. 2:**
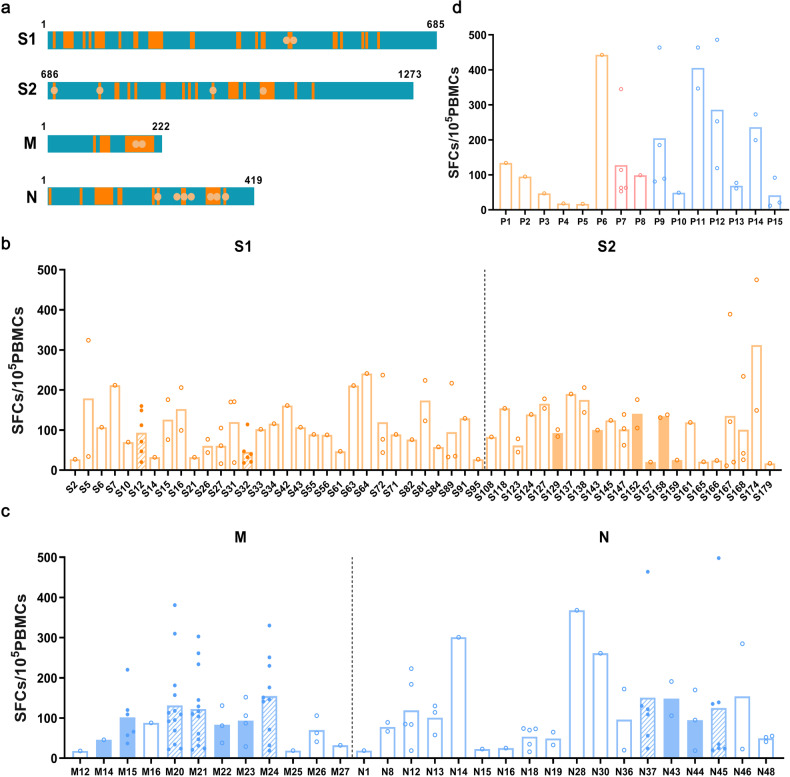
T cell responses of COVID-19 convalescents to identified immunogenic SARS-CoV-2 overlapping peptides and epitopes. **a** The distribution of 83 positive overlapping peptides and 15 CD8^+^ T cell epitopes on different proteins of SARS-CoV-2. Orange rectangles represent positive overlapping peptides and orange circles represent positive CD8^+^ T cell epitopes. T cell responses of COVID-19 convalescents to 83 positive overlapping peptides from the S (**b**), M and N (**c**) proteins. Data are presented as mean. T cell responses to 8 immunodominant overlapping peptides are represented in oblique line-filled circles. T cell responses to the 12 conserved overlapping peptides are represented in filled bars. **d** T cell responses of COVID-19 convalescents to 15 positive epitopes. Data are presented as mean. T cell responses to epitopes from S, M and N proteins are represented in orange, red and blue colors, respectively

PBMCs were expanded using 4 SARS-CoV-2 peptide pools (S1, S2, M and N) and then stimulated using two- and three-dimensional matrix peptide pools in COVID-19 convalescents (*n* = 32) from Macheng at 6 m in July 2020. For positive peptide pools, PBMCs were then stimulated with single peptides. A total of 83 positive overlapping peptides, including 53 from the S protein (31 from S1 and 22 from S2), 12 from M, and 18 from N (Fig. 2a, Supplementary Table 1), were recognized by PBMCs stimulated after expansion. The T cell response stimulated by S174 was the strongest in S peptide pool, which was up to 475 SFCs/10^5^ PBMCs. The T cell response stimulated by N45 was the strongest and up to 498 SFCs/10^5^ PBMCs in M and N peptide pool. Strikingly, 8 dominant overlapping peptides were recognized by 6 or more of the 32 COVID-19 convalescents. S12, S32, M15 and N37 were recognized by 19% (6/32) of the COVID-19 convalescents. There were more overlapping peptides with high response frequency in the M and N proteins. The percentages of COVID-19 convalescents showing positive T cell responses were 41% (13/32) for M20 and M21, 31% (10/32) for M24, and 22% (7/32) for N45 (Fig. 2b, c).

To further identify SARS-CoV-2 CD8^+^ T cell epitopes, we predicted a total of 97 short peptides (8- to 11-mer) within the 83 positive overlapping peptides (Supplementary Table 2). First, we predicted 75 short peptides according to the HLA alleles of the COVID-19 convalescents using NetMHCpan-4.0/4.1. Then, we summarized the published SARS-CoV epitopes using IEDB and then predicted 22 short peptides at the same position with SARS-CoV-2. A total of 15 SARS-CoV-2 CD8^+^ T cell epitopes were identified, including 6 on the S protein, 2 on the M and 7 on the N (Fig. 2a, Table 1). These epitopes were linked to eight different HLA restrictions, based on predicted HLA binding capacities matched to the HLA alleles of the COVID-19 convalescents.

**Table 1:** The identified SARS-CoV-2 CD8^+^ T cell epitopes

Participants	Name	Protein (position)	SARS-CoV-2 amino acid sequence	HLA restriction
M-60	P1	S (448−456)	NYNYLYRLF	A2402
M-92	P2	S (456−464)	FRKSNLKPF	B2704
M-21	P3	S (687−695)	VASQSIIAY	B1501
M-41	P4	S (753−761)	LLQYGSFCT	A0201
M-21	P5	S (962−970)	LVKQLSSNF	B1501
M-13	P6	S (1042−1050)	VVFLHVTYV	A0206
M-3/25/63/71/90	P7	M (171−180)	ATSRTLSYYK	A1101
M-71	P8	M (171−179)	ATSRTLSYY	B5801
M-9/16/19/59	P9	N (219−227)	LALLLLDRL	A0201
M-1	P10	N (265–274)	TKAYNVTQAF	B2704
M-1/95	P11	N (266–274)	KAYNVTQAF	B2704
M-1/44/48	P12	N (267–274)	AYNVTQAF	A2402
M-27/32	P13	N (316−324)	GMSRIGMEV	A0201
M-11/19	P14	N (322−331)	MEVTPSGTWL	B4001
M-11/14/ 32	P15	N (361−369)	KTFPPTEPK	A1101

### Nonconserved overlapping peptides dominate the cross-T cell responses between SARS-CoV-2 and SARS-CoV compared to conserved peptides

**Fig. 3:**
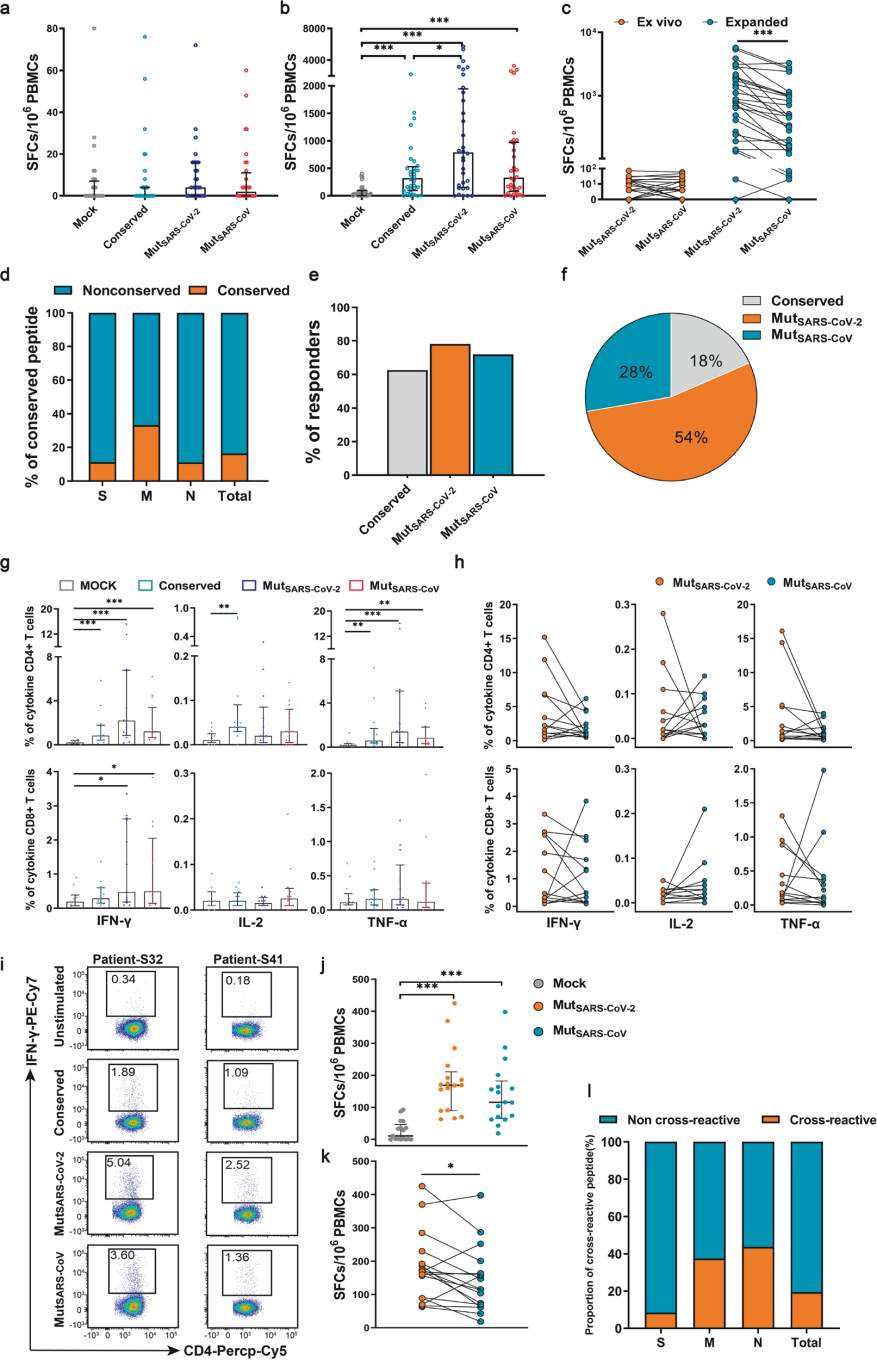
Cross-T cell responses to the conserved and nonconserved overlapping peptides in COVID-19 convalescents. PBMCs of COVID-19 convalescents (*n* = 32) were stimulated with the Conserved-pool, Mut_SARS-CoV-2_-pool, and Mut_SARS-CoV_-pool directly ex vivo (**a**) or after an expansion in vitro (**b**). **c** The changes in T cell responses of individual convalescents to Mut_SARS-CoV-2_-pool and Mut_SARS-CoV_-pool directly ex vivo (left) or after an expansion in vitro (right). It shows the same results as **a** and **b**. **d** The proportions of the conserved and nonconserved peptides in different proteins. **e** The percentages of COVID-19 convalescents with positive T cell responses to the Conserved-pool, Mut_SARS-CoV-2_-pool and Mut_SARS-CoV_-pool. **f** The proportions of T cell responses to different peptide pools at the individual level by calculating the average of 32 convalescents. **g** The percentages of T cells (secreting IFN-γ, IL-2, and/or TNF-α) specific for different peptide pools in the 13 COVID-19 convalescents. **h** The changes in T cells (secreting IFN-γ, IL-2, and/or TNF-α) specific for Mut_SARS-CoV-2_-pool and Mut_SARS-CoV_-pool. It shows the same results as **g**. **i** The examples of CD4^+^ T cells (secreting IFN-γ) specific for different peptide pools. The controls were not stimulated with peptides. T cell responses to the 14 pairs of overlapping peptides with cross-reaction in 13 COVID-19 convalescents (**j**) and paired comparison (**k**). **l** The proportions of cross-reactive overlapping peptides on different proteins. Data are presented as median (IQR) or N (%). A Mann–Whitney U-test was used in **a**, **b**, **g**, and **j**. A Wilcoxon matched-pairs signed rank test was used in **c**, **h**, and **k**. Two-tailed *P* values were calculated. *P < 0.05; **P < 0.01; ***P < 0.001

Based on our identified 83 positive overlapping SARS-CoV-2 peptides, we designed the conserved pool (Conserved-pool) and nonconserved pools (including Mut_SARS-CoV-2_-pool and Mut_SARS-CoV_-pool) (Supplementary Table 1). The antigenicity changes of the nonconserved peptides at the peptide pool level were verified in COVID-19 convalescents. There was no significant difference between the T cell responses to 3 different peptide pools when freshly-isolated PBMCs were stimulated ex vivo (*P* > 0.05) (Fig. 3a). Then, PBMCs were expanded with the peptide pool including 83 positive overlapping peptides of SARS-CoV-2 and stimulated with the conserved pool and nonconserved pools. After in vitro expansion, the Mut_SARS-CoV-2_-pool induced higher T cell responses compared with the Conserved-pool (*P* < 0.05) (Fig. 3b). The T cell responses were higher to the Mut_SARS-CoV-2_-pool compared to the Mut_SARS-CoV_-pool for each individual among most COVID-19 convalescents after pairing comparison (*P* < 0.05) (Fig. 3c). Apparently, the nonconserved peptides accounted for a higher proportion in each protein compared with the conserved peptides (Fig. 3d). This indicates that the nonconserved peptides may contribute more to the cross-reaction between these two viruses. Overall, 63% (20/32) of COVID-19 convalescents showed positive T cell responses for the Conserved-pool, 78% (25/32) for Mut_SARS-CoV-2_-pool, and 72% (23/32) for Mut_SARS-CoV_-pool, which were higher to nonconserved peptides (Fig. 3e). For each convalescent, the T cell response to SARS-CoV-2 (72%) was higher than the response to SARS-CoV (46%) (Fig. 3f).

Intracellular cytokine staining was also performed to confirm specific T cell responses to different peptide pools. The results showed that there were IFN-γ-producing SARS-CoV-reactive CD4^+^ and CD8^+^ T cells in the COVID-19 convalescents (Fig. 3g–i). Besides, the Conserved-pool-specific CD8^+^ T cells were low and there was no difference with the negative control (Fig. 3g). In most COVID-19 convalescents, no significant difference was observed between the levels of CD4^+^ and CD8^+^ T cells specific for the Mut_SARS-CoV-2_-pool and the Mut_SARS-CoV_-pool (*P* > 0.05) (Fig. 3h). This further confirms the importance of the nonconserved peptides in cross-recognition of SARS-CoV by the COVID-19 convalescents.

Subsequently, after incubation with Mut_SARS-CoV-2_-pool, PBMCs from COVID-19 convalescents were stimulated by each single nonconserved peptide from Mut_SARS-CoV-2_-pool and Mut_SARS-CoV_-pool. Ultimately, 14 pairs of overlapping peptides which could induce cross-reactivity were obtained in the COVID-19 convalescents (Table 2). Similar to the above results, most COVID-19 convalescents had decreased T cell responses to the overlapping peptides from SARS-CoV-2 to SARS-CoV based on pairing comparison (*P* < 0.05) (Fig. 3j, k). The cross-reactive peptides only accounted for a small part of the nonconserved peptides and the number of cross-reactive peptides of N was more than that of S and M (Fig. 3l). This may be the reason why the N protein could induce stronger cross-reactivity and there was no significant difference between the T cell responses to the N proteins of these two viruses.

**Table 2:** The nonconserved overlapping peptides that are cross-reactive

Participants	Name	Protein (position)^a^	SARS-CoV-2 amino acid sequence	SARS-CoV amino acid sequence
M-11	S89	S (555–570)	SNKKFLPFQQFGRDIA	S**S**K**R**F**Q**PFQQFGRD**VS**
M-10	S123	S (780–797)	EVFAQVKQIYKTPPIKDF	EVFAQVKQ**M**YKTP**TL**K**Y**F
M-10	S127	S (801–818)	NFSQILPDPSKPSKRSFI	NFSQILPDP**L**KP**T**KRSFI
M-10	S147	S (921–938)	KLIANQFNSAIGKIQDSL	K**Q**IANQFN**K**AI**SQ**IQ**E**SL
M-34/84	M20	M (141–158)	GAVILRGHLRIAGHHLGR	GAVIIRGHLR**M**AGH**S**LGR
M-34	M21	M (149–166)	LRIAGHHLGRCDIKDLPK	LR**M**AGH**S**LGRCDIKDLPK
M-17	M24	M (172–188)	TSRTLSYYKLGASQRVA	TSRTLSYYKLGASQRV
M-82	N13	N (86–102)	YYRRATRRIRGGDGKMK	YYRRATRR**V**RGGDGKMK
M-26	N18	N (124–139)	GANKDGIIWVATEGAL	GANK**E**GI**V**WVATEGAL
M-26	N19	N (130–146)	IIWVATEGALNTPKDHI	I**V**WVATEGALNTPKDHI
M-30	N30	N (210–227)	MAGNGGDAALALLLLDRL	MA**SG**GG**ET**ALALLLLDRL
M-95	N37	N (261–277)	KRTATKAYNVTQAFGRR	KRTATK**Q**YNVTQAFGRR
M-8/45	N45	N (321–338)	GMEVTPSGTWLTYTGAIK	GMEVTPSGTWLTY**H**GAIK
M-18/89	N48	N (344–361)	PNFKDQVILLNKHIDAYK	P**Q**FKD**N**VILLNKHIDAYK

^a^The position of amino acid is numbered according to the reference sequence of SARS-CoV-2

### CD8^+^ T cell cross-reactivity between SARS-CoV-2- and SARS-CoV-derived peptides

**Fig. 4:**
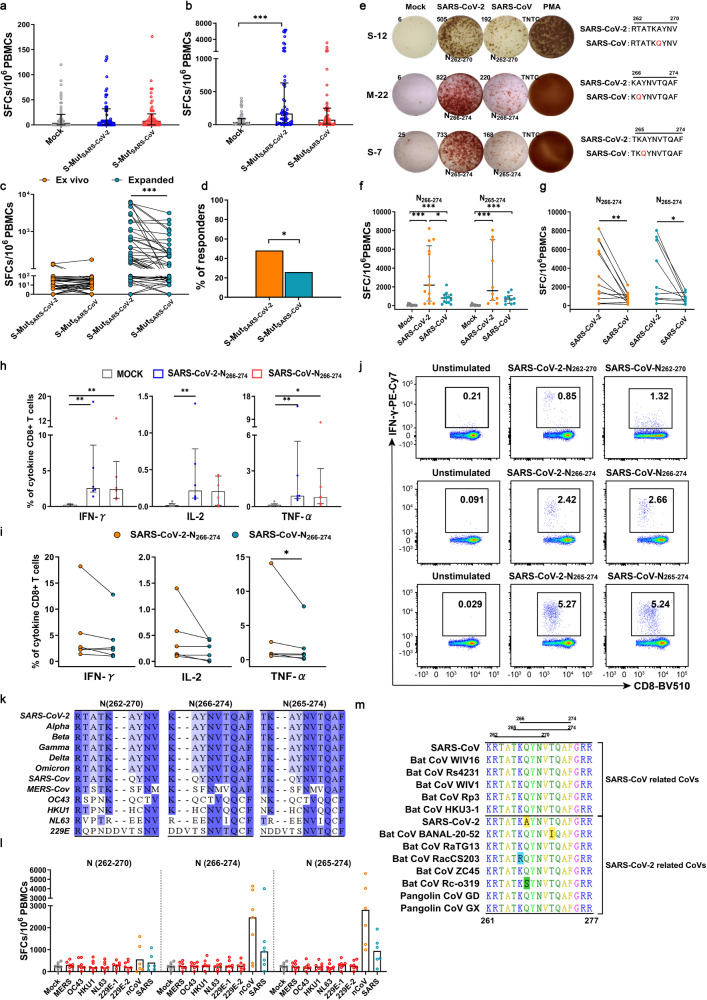
Cross-CD8^+^ T cell responses to SARS-CoV-2- and SARS-CoV-derived short epitopes. PBMCs of COVID-19 convalescents (*n* = 54) were stimulated with S-Mut_SARS-CoV-2_-pool and S-Mut_SARS-CoV_-pool directly ex vivo (**a**) or after an expansion in vitro (**b**). **c** The comparison of T cell responses within individuals to S-Mut_SARS-CoV-2_-pool and S-Mut_SARS-CoV_-pool directly ex vivo (left) or after an expansion in vitro (right). It shows the same results as **a** and **b**. **d** The percentages of COVID-19 convalescents with positive T cell responses to S-Mut_SARS-CoV-2_-pool and S-Mut_SARS-CoV_-pool. **e** The representative ELISpot results of 3 pairs of cross-reactive peptides after an expansion in vitro. The location and sequence of peptides are shown. PMA represented the phorbol ester which was used as a positive control. TNTC represented too many to count. T cell responses to SARS-CoV-2-N_266-274_ and SARS-CoV-N_266-274_ (*n* = 13) and SARS-CoV-2-N_265-274_ and SARS-CoV-N_265-274_ (*n* = 10) (**f**) and the changes between different peptides (**g**). They shows the same results. The percentages of CD8^+^ T cells (secreting IFN-γ, IL-2, and/or TNF-α) specific for SARS-CoV-2-N_266-274_ and SARS-CoV-N_266-274_ (**h**) and the changes in CD8^+^ T cells specific for SARS-CoV-2-N_266-274_ and SARS-CoV-N_266-274_ (**i**). They shows the same results. **j** The examples of CD8^+^ T cells (secreting IFN-γ) specific for the 3 pairs of cross-reactive peptides. The controls were not stimulated with peptides. **k** The alignment of the SARS-CoV-2-N_262-270_, -N_266-274_, and -N_265-274_ amino acid sequences in SARS-CoV-2 variants and other human coronaviruses. **l** T cell responses to short peptides of other human coronaviruses at the same position. **m** The alignment of the overlapping peptide (N261-277) amino acid sequences in SARS-CoV related CoVs and SARS-CoV-2 related CoVs. Data are presented as median (IQR) or N (%). A Mann–Whitney U-test was used in **a**, **b**, **f**, and **h**. A Wilcoxon matched-pairs signed rank test was used in **c**, **g**, and **i**. A chi-square test was used for **d**. Two-tailed *P* values were calculated. *P < 0.05; **P < 0.01; ***P < 0.001

To identify cross-reactive CD8^+^ T cell epitopes, we synthesized 20 pairs of short peptides among the cross-reactive overlapping peptides according to the Immune Epitope Database. These short peptides were classified into S-Mut_SARS-CoV-2_-pool and S-Mut_SARS-CoV_-pool (Table 3). Similarly, the two peptide pools were used to stimulate PBMCs from COVID-19 convalescents either directly ex vivo or after an in vitro expansion with the peptide pool including 14 cross-reactive overlapping peptides of SARS-CoV-2 (Fig. 4a–c). There were 48% (26/54) and 26% (14/54) of COVID-19 convalescents with positive CD8^+^ T cell responses for S-Mut_SARS-CoV-2_-pool and S-Mut_SARS-CoV_-pool, respectively, after the expansion (Fig. 4d).

**Table 3:** The nonconserved CD8^+^ T cell epitopes composing the Mut_SARS-CoV-2_-pool and Mut_SARS-CoV_-pool

Name	amino acid sequence	Protein (position)	SARS-CoV-2 epitope sequence	SARS-CoV epitope sequence
S89	SNKKFLPFQQFGRDIA	S (558–567)	KFLPFQQFGR	**R**F**Q**PFQQFGR
S123	EVFAQVKQIYKTPPIKDF	S (780–788)	EVFAQVKQI	EVFAQVKQ**M**
		S (780–789)	EVFAQVKQIY	EVFAQVKQ**M**Y
		S (781–789)	VFAQVKQIY	VFAQVKQ**M**Y
		S (786–797)	KQIYKTPPIKDF	KQ**M**YKTP**TL**K**Y**F
		S (788–797	IYKTPPIKDF	**M**YKTP**TL**K**Y**F
S127	NFSQILPDPSKPSKRSFI	S (805–814)	ILPDPSKPSK	ILPDP**L**KP**T**K
		S (805–813)	ILPDPSKPS	ILPDP**L**KP**T** ^a^
S147	KLIANQFNSAIGKIQDSL	S (923–931)	IANQFNSAI	IANQFN**K**AI
M20	GAVILRGHLRIAGHHLGR	M (142–150)	AVILRGHLR	AVI**I**RGHLR
		M (148–158)	HLRIAGHHLGR	HLR**M**AGH**S**LGR
M21	LRIAGHHLGRCDIKDLPK	M (150–158)	RIAGHHLGR	R**M**AGH**S**LGR
N13	YYRRATRRIRGGDGKMK	N (86–95)	YYRRATRRIR	YYRRATRR**V**R
		N (93–101)	RIRGGDGKM	R**V**RGGDGKM
		N (93–102)	RIRGGDGKMK	R**V**RGGDGKMK
N19	IIWVATEGALNTPKDHI	N (130–138)	IIWVATEGA	I**V**WVATEGA
N30	MAGNGGDAALALLLLDRL	N (215–224)	GDAALALLLL	G**ET**ALALLLL ^a^
N37	KRTATKAYNVTQAFGRR	N (262–270)	RTATKAYNV	RTATK**Q**YNV
		N (266–274)	KAYNVTQAF	K**Q**YNVTQAF
		N (265–274)	TKAYNVTQAF	TK**Q**YNVTQAF ^a^

^a^These three pairs of peptides were synthesized with reference to the epitopes of SARS-CoV, and the other pairs referred to the epitopes of SARS-CoV-2

Meanwhile, for positive peptide pools, we also stimulated PBMCs with each single short peptide from the two pools and obtained 3 pairs of short peptides that had cross-reactivity (Fig. 4e). Interestingly, these short peptides were on the same overlapping peptide (N261-277) in the N protein and had the same amino acid substitution (A267Q) at either anchoring or exposed residues. There were 13 COVID-19 convalescents responding to the SARS-CoV-2-N_266-274_ and SARS-CoV-N_266-274_ peptides, while 10 COVID-19 convalescents responding to the SARS-CoV-2-N_265-274_ and SARS-CoV-N_265-274_ peptides (Fig. 4f, g). The peptide dose-response relationship of SARS-CoV-2-N_266-274_ and SARS-CoV-N_266-274_ was also analyzed (Supplementary Fig. 1a). Subsequently, we further tested the ability of the cross-reactive short peptides to activate CD8^+^ T cells by using intracellular cytokine staining. The single peptide-specific CD8^+^ T cells that produced IFN-γ and TNF-α were found (Fig. 4h–j).

We investigated the sequence conservation of peptides within the corresponding sequences of SARS-CoV-2 BA.1-5 and other human coronaviruses. The results of the sequence alignment showed that these peptide sequences were conserved in SARS-CoV-2 variants, but varied in other human coronaviruses (Fig. 4k). We synthesized 18 short peptides of other human coronaviruses and tested their immunogenicity. However, no positive responses to these short peptides were found except for the two viruses within human coronaviruses (Fig. 4l). Besides, we also found the sequence of the overlapping peptide (N261-277) was conserved in SARS-CoV related CoVs (Fig. 4m).

### The HLA I binding of cross-reactive CD8^+^ T cell epitopes

**Fig. 5:**
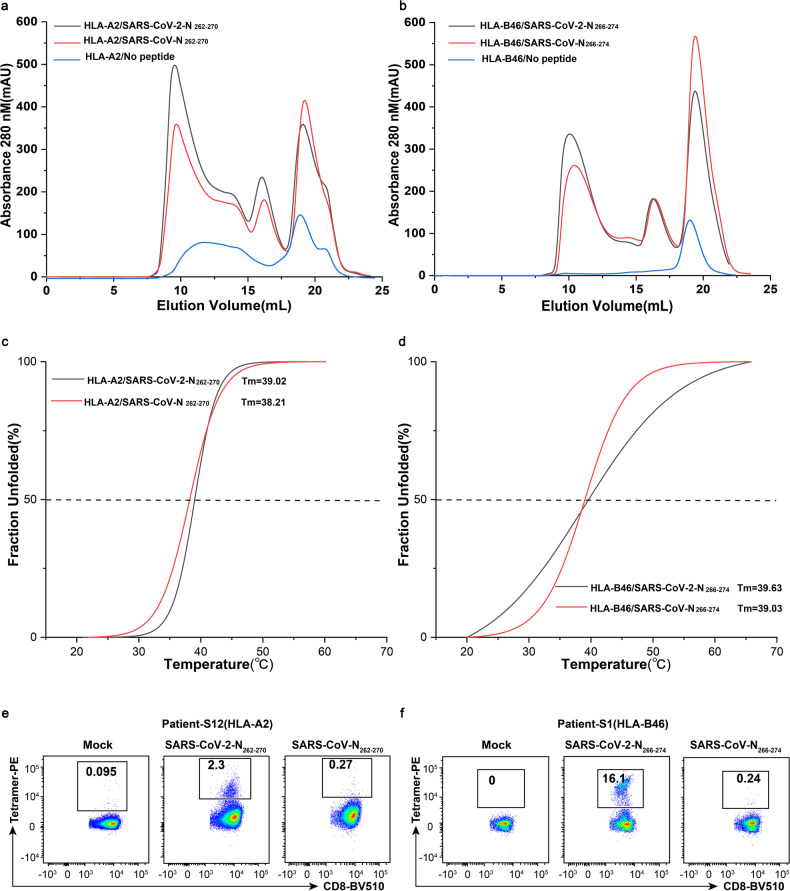
The presentation of cross-reactive peptides by HLA-A2 and HLA-B46. **a** The results of in vitro refolding of the SARS-CoV-2-N_262-270_ (black), SARS-CoV-N_262-270_ (red), and no peptide (blue, negative control) with HLA-A2. **b** The results of in vitro refolding of the SARS-CoV-2-N_266-274_ (black), SARS-CoV-N_266-274_ (red), and no peptide (blue, negative control) with HLA-B46. **c** The thermal stability of the HLA-A2/SARS-CoV-2-N_262-270_ (black) and HLA-A2/SARS-CoV-N_262-270_ (red) complexes. **d** The thermal stability of the HLA-B46/SARS-CoV-2-N_266-274_ (black) and HLA-B46/SARS-CoV-N_266-274_ (red) complexes. **e** The example of SARS-CoV-2-specific CD8^+^ T cells stained by HLA-A*0201 tetramers prepared with the SARS-CoV-2-N_262-270_ and SARS-CoV-N_262-270_ peptides. **f** The example of SARS-CoV-2-specific CD8^+^ T cells stained by HLA-B*4601 tetramers prepared with the SARS-CoV-2-N_266-274_ and SARS-CoV-N_266-274_ peptides. The controls were stained with an irrelevant tetramer

Next, we investigated the HLA restrictions of cross-reactive epitopes according to HLA typing of positive convalescents, together with the peptide binding motif predicted by NetMHCpan-4.0/4.1. The results showed that the SARS-CoV-2-N_266-274_ and SARS-CoV-N_266-274_ peptides could bind to 10 different HLA-I alleles (Table 4). To verify these cross-reactive epitopes, we performed in vitro refolding of the SARS-CoV-2-N_262-270_ and SARS-CoV-N_262-270_ with HLA-A2 (Fig. 5a), the SARS-CoV-2-N_266-274_ and SARS-CoV-N_266-274_ with HLA-B46 (Fig. 5b), and the SARS-CoV-2-N_265-274_ and SARS-CoV-N_265-274_ with HLA-B46 (Supplementary Fig. 1b). We found that the SARS-CoV-2-N_262-270_, SARS-CoV-N_262-270_, SARS-CoV-2-N_266-274_ and SARS-CoV-N_266-274_ peptides were successfully refolded with HLA molecules. The peaks of the HLA/peptide complexes were similar for peptides from these two viruses, which showed a similar binding capacity. Circular dichroism was used to assess the thermal stability of the HLA/peptide complexes, reflecting their binding ability and stability.As shown in Fig. 5c, the melting temperature of HLA-A2/SARS-CoV-N_262-270_ was 38.21 °C and it was slightly lower than that of the HLA-A2/SARS-CoV-2-N_262-270_ at 39.02 °C. Similarly, the melting temperature of HLA-B46/SARS-CoV-N_266-274_ only decreased by 0.6 °C compared with that of HLA-B46/SARS-CoV-2-N_266-274_ (Fig. 5d). This indicates that when Ala is mutated into Gln (position 2 or 6), the binding ability between the peptides and HLA molecules has no significant changes.

**Table 4:** The HLA restrictions of cross-reactive CD8^+^ T cell epitopes

Name	Epitope sequence	Published HLA restriction	Predicted HLA restriction	Count^a^
SARS-CoV-2-N_262-270_	RTATKAYNV	A*02:01^30^	A*02:06	2
SARS-CoV-N_262-270_	RTATKQYNV			
SARS-CoV-2-N_266-274_	KAYNVTQAF			
			A*32:01	1
		B*35:01^31^	B*13:01	3
		B*57:01^31,32^	B*13:02	1
		B*51:01^32^	B*37:01	1
			B*48:01	1
SARS-CoV-N_266-274_	KQYNVTQAF	A*31:01	B*52:01	3
		A*24:02^33^	A*24:02	1
		B*27:05	B*27:04	1
		B*46:01	B*46:01	3
		B*58:01	B*58:01	2
		B*15:01^33^		
SARS-CoV-2-N_265-274_	TKAYNVTQAF		B*46:01	1
SARS-CoV-N_265-274_	TKQYNVTQAF	B*15:25^34^		

^a^The numbers represent the frequency of allele occurrence

Subsequently, we also prepared HLA/peptide tetramers composed of peptides bound to the HLA-A*0201 and HLA-B*4601 molecules. When PBMCs from COVID-19 convalescents with positive responses to these peptides were used, tetramers-specific CD8^+^ T cells were both detected for these two viruses, though with a lower proportion for SARS-CoV (Fig. 5e, f).

### The TCR repertoires for recognizing the SARS-CoV-2-N_262-270_ and SARS-CoV-N_262-270_ peptides are different

**Fig. 6:**
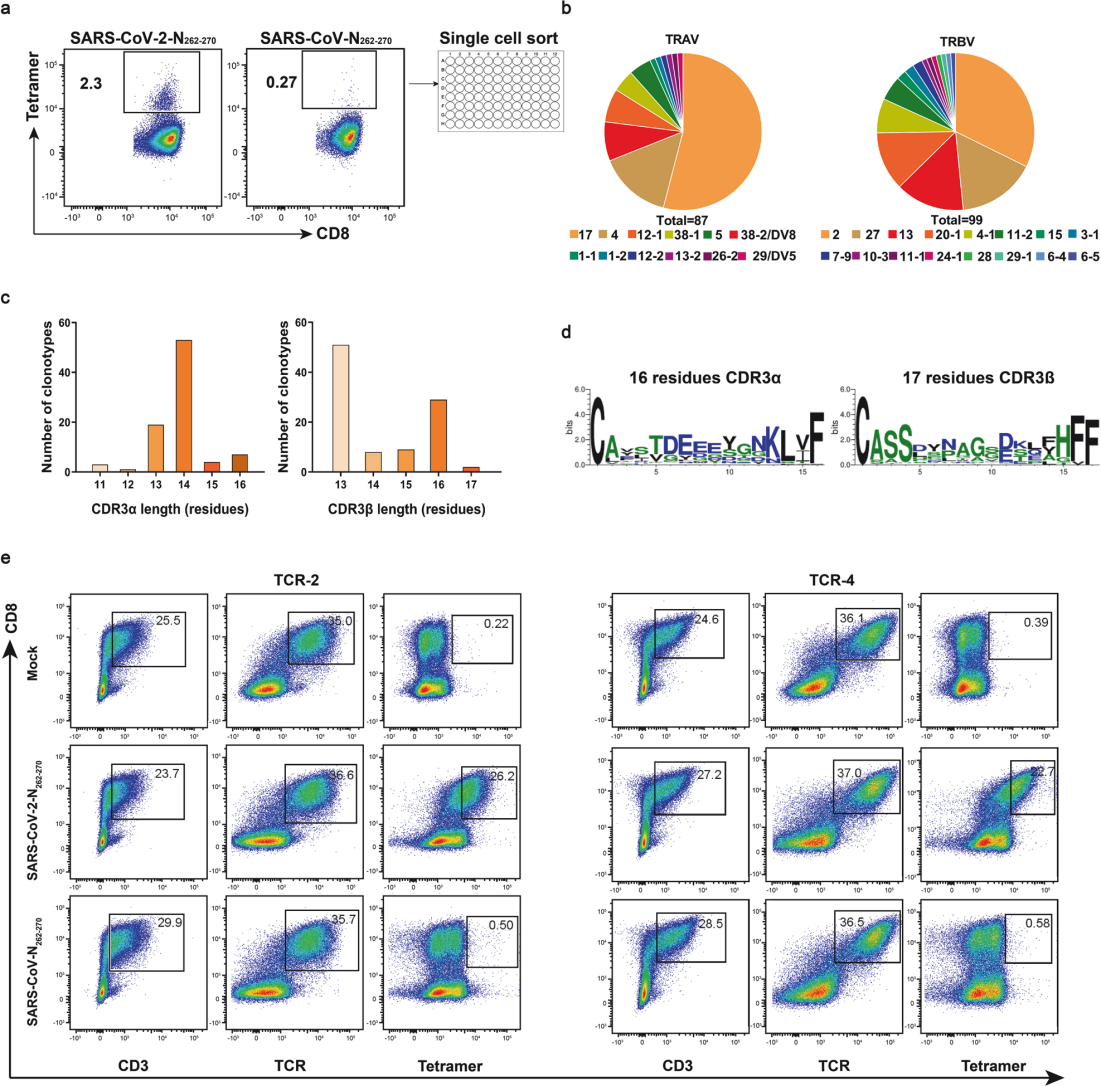
The TCR repertoires recognizing peptides SARS-CoV-2-N_262-270_ and SARS-CoV-N_262-270_. **a** Schematic diagram illustrating the representative tetramer staining for single-cell isolation and TCR sequencing of the SARS-CoV-2-N_262-270_ and SARS-CoV-N_262-270_-specific T cells after an expansion in vitro. **b** The pie chart displaying the preferred TRAV (left) and TRBV (right) usage of the SARS-CoV-2-N_262-270_-specific TCRs. **c** The summary of CDR3α (left) and CDR3β (right) lengths for SARS-CoV-2-N_262-270_-specific TCR clonotypes. **d** The motif of the CDR3α (left) and CDR3β (right) loops from a COVID-19 convalescent. **e** The in vitro expression of the TCR-2 and TCR-4 pairs and their binding to tetramers from SARS-CoV-2 and SARS-CoV

To further explore the mechanism of cross-reaction and to characterize specific CD8^+^ T cells, we detected TCR repertoires for the SARS-CoV-2-N_262-270_ and SARS-CoV-N_262-270_ peptides. Tetramers-specific CD8^+^ T cells were sorted from one COVID-19 convalescent by fluorescence-activated cell sorting and sequenced by single-cell TCR sequencing (Fig. 6a). For SARS-CoV-2-N_262-270_, 87 sequences of α chain and 99 sequences of β chain were obtained. For SARS-CoV-N_262-270_, 58 sequences of α chain and 105 sequences of β chain were obtained (Supplementary Table 3). However, we only identified strong usage biases of T cell receptor alpha variable region (TRAV) and T cell receptor beta variable region (TRBV) in SARS-CoV-2-N_262-270_-specific T cells, with 54 and 32% of these single cells expressing the TRAV17 and TRBV2 gene, respectively (Fig. 6b). We also observed a typical distribution in complementarity determining region 3 alpha (CDR3α) length and a preference for 14 amino acids (Fig. 6c) and the sequences of the CDR3 loop of TRAV showed preference of negative residues for the CDR3α (Fig. 6d) in 87 sequences of α chain. We also obtained 15 unique αβ TCR pairs (Table 5), and the pair with the highest frequency (48%) was exactly TRAV17 and TRBV2.

**Table 5:** The TCR pairs of SARS-CoV-2-N_262-270_-specific CD8^+^ T cells in COVID-19 convalescents

Number	TRAV	CDR3α	TRAJ	TRBV	CDR3β	TRBD	TRBJ	Count	Freq%
1	17*01	CATDEEEYGNKLVF	47*01	2*01	CASSDYNAEKLFF	1*01	1-4*01	26	48.15
2	4*01	CLVGDVDTDKLIF	34*01	20-1*05	CSASHPGDSPLHF	2*01	1-6*01	7	12.96
3	38-2/DV8*01	CAYSYGGSQGNLIF	42*01	13*01	CASSSLTGGDEQYV	1*01	2-7*02	4	7.41
4	17*01	CATDEDTNAGKSTF	27*01	4-1*01	CASSPDPWGVDTEAFF	2*01	1-1*01	3	5.56
5	12-1*01	CVVLMDSSYKLIF	12*01	27*01	CASSLSPAGSDSEAFF	1*01	1-1*01	3	5.56
6	5*01	CAESIGYGGSQGNLIF	42*01	13*01	CASSLQNQETQYF	--	2-5*01	2	3.70
7	4*01	CLVEKEEGGKLIF	23*01	20-1*05	CSAPGLAGSTDTQYF	2*01	2-3*01	1	1.85
8	17*01	CATDADNFNKFYF	21*01	28*01	CASTLRGVREQFF	1*01	2-1*01	1	1.85
9	17*01	CATDAGLTGGGNKLTF	10*01	3-1*01	CASSQELALFNEQFF	2*01	2-1*01	1	1.85
10	1-2*01	CAVSNFNKFYF	21*01	3-1*01	CASRGERGYEQYF	2*01	2-7*01	1	1.85
11	12-1*01	CVVEMDSSYKLIF	12*01	27*01	CASSLSRAGGDSPLHF	1*01	1-6*01	1	1.85
12	26-2*01	CILRPQEETSGSRLTF	58*01	13*01	CASSKGVKENTEAFF	1*01	1-1*01	1	1.85
13	38-1*03	CAFIVPQGGSEKLVF	57*01	15*02	CATSDYNRDFYGYTF	2*01	1-2*01	1	1.85
14	29/DV5*01	CAPTNGNKLVF	47*01	7-9*03	CASSPGVQYEQYF	1*01	2-7*01	1	1.85
15	38-2/DV8*01	CASVDSNYQLIW	33*01	11-1*01	CASSPGPGTIDTDTQYF	2*01	2-3*01	1	1.85

Then, we selected TCR-1 and -2 pairs from the 15 unique αβ TCR pairs according to the frequency of the TCR of SARS-CoV-2-N_262-270_, and selected TCR-4 and -7 pairs according to the similarity with the TCR of SARS-CoV-N_262-270_ for in vitro function verification. We transfected 293T cells with CD3, CD8 and TCR plasmids, and verified their expression and binding to tetramers by flow cytometry. The results showed that the TCR-2 and TCR-4 pairs had well defined binding with SARS-CoV-2-N_262-270_ tetramer, but had no obvious binding with SARS-CoV-N_262-270_ tetramer (Fig. 6e). However, both the TCR-1 and TCR-7 pairs were weakly bound to two tetramers (Supplementary Fig. 1c). No common TCR repertoires between SARS-CoV-2-N_262-270_- and SARS-CoV-N_262-270_-specific T cells were found, which may indicate that although peptides within this position of these two viruses are cross-reactive, but their TCR repertoires are peptide-specific.

## Discussion

To defend against the ongoing pandemic of SARS-CoV-2 and the potential emerging human coronaviruses, taking preventive measures, especially vaccine inoculation, is critical. The cellular immunity after SARS-CoV-2 infection or vaccination lasts longer and has great potential to induce cross-reactivity with other coronaviruses. Moreover, an established adaptive immunity towards closely related viruses can reduce susceptibility.^34,35^ Therefore, this study aims to investigate whether PBMCs from COVID-19 convalescents have cross-reactivity against SARS-CoV and to identify relatively conserved epitopes of SARS-CoV-2 and SARS-CoV. Our findings may contribute to the design of peptide-based universal vaccines against coronaviruses.^36^

SARS-CoV-2-specific T cells from COVID-19 convalescents at 6 and 12 months were cross-reactive to SARS-CoV, showing stronger cross-reactivity to the N protein of SARS-CoV. This may be because the sequences of the N are highly conserved and the cross-reactive peptides of N are more than those of S and M proteins. Moreover, we rationally designed the conserved and nonconserved peptide pools and assessed their roles in cellular immune responses. Interestingly, the nonconserved peptides could induce higher T cell responses than the conserved peptides, which is different from the influenza virus.^37^ This may imply that the nonconserved peptides deserve more attention in the study of cross-immunity of coronaviruses. Compared to SARS-CoV, the T cell responses of most COVID-19 convalescents responded higher to the peptide pools of SARS-CoV-2. Due to limited conditions, we did not recruit SARS convalescents. However, a previous study showed that the T cell responses were decreased from SARS-CoV to SARS-CoV-2 in most SARS convalescents.^32^

We also observed positive T cell responses against SARS-CoV-2 and SARS-CoV in unexposed people. This finding is in line with previous reports on preexisting SARS-CoV-2-reactive T cells in HCs.^38–40^ Our results showed that the T cell responses of HCs to different peptide pools of SARS-CoV were slightly lower than those to peptide pools of SARS-CoV-2. The reason may be that PBMCs of HCs were stimulated with the peptide pools of SARS-CoV-2 for 9 days. The results also indicated that the S2 protein of both viruses induced higher T cell responses than the S1 protein. This might be due to the C-terminal epitopes in the S protein having greater homology to common cold coronavirus compared to the N-terminal epitopes.^41^

Besides, in COVID-19 convalescents, we found a significant linear correlation between disease severity during the acute phase and the levels of cross-recognition to SARS-CoV. This may be explained by the relationship between disease severity during the acute phase and the immunological memory to SARS-CoV-2. Several studies have shown that T cell responses tend to be higher following symptomatic infection than asymptomatic infection.^42,43^ However, the specific duration of this cross-recognition needs further observation.

Then, we predicted peptides with NetMHCpan-4.0/4.1 and identified 83 positive overlapping peptides and 15 CD8^+^ T cell epitopes of SARS-CoV-2. NetMHCpan’s core algorithm, NNAlign_MA, is built on large and diverse datasets, covering both class I and class II data. This method consistently outperformed state-of-the-art techniques, enhancing allele coverage for accurate predictions and improving the identification of both eluted ligands and T cell epitopes.^44,45^ In the positive overlapping peptides, 71 nonconserved peptides far exceeded 12 conserved peptides and there were 14 nonconserved peptides with cross-reactivity. If T cell responses are detected in a wider population, there will inevitably be more cross-reactive peptides due to the involvement of more HLA-I or -II subtypes.

Then we identified 3 pairs of cross-reactive CD8^+^ T cell epitopes in COVID-19 convalescents. And they are on the same overlapping peptide (N261-277) in N protein and the SARS-CoV-2-N_266-274_ and SARS-CoV-N_266-274_ were positive in 13 COVID-19 convalescents, associated with 10 different HLA allele restrictions. Through the peptide binding motif analyses of these potential SARS-CoV-2-N_266-274_-restricted HLA alleles, alleles HLA-B*46, B*52…have overlapping binding peptide motif, which may indicate that individuals with these HLA alleles may all respond to this dominant peptide SARS-CoV-2-N_266-274_ and its corresponding peptide SARS-CoV-N_266-274_. Meanwhile, SARS-CoV related CoVs may still cause new infections in the future.^46^ We found the sequence of the overlapping peptide (N261-277) is conserved in different SARS-CoV-2 variants and SARS-CoV related CoVs, suggesting that it can be used as a candidate peptide for developing universal vaccines against Sarbecoviruses.

Circular dichroism (CD) spectrometry, a widely used method for detecting the changes of protein structure and conformation,^47^ showed that the binding capacities with HLA were similar for peptides from SARS-CoV-2 and SARS-CoV. Furthermore, we identified biased TRAV and TRBV usages in SARS-CoV-2-N_262-270_-specific T cells and no common TCR repertoires between SARS-CoV-2-N_262-270_- and SARS-CoV-N_262-270_-specific T cells were found. This may indicate that although peptides within this position of SARS-CoV-2 and SARS-CoV are cross-reactive, but their TCR repertoires are peptide-specific. One reason may be that the proportion of SARS-CoV-N_262-270_-specific CD8^+^ T cells in sorted cells is too low to identify their specific TCR. Another reason could be that the primary anchor residues of HLA-A2 restricted epitopes are typically two amino acids with hydrophobic side chains. These residues fit into the hydrophobic peptide-binding clefts of HLA-A2 at position 2 of the N-terminus and at the C-terminus.^17,48^ While, the amino acids in position 6 of the N-terminus act as secondary anchor residues of the nonamer epitopes. Thus, the amino acid substitution (A267Q) between the SARS-CoV-2-N_262-270_ and SARS-CoV-N_262-270_ peptides in position 6 may also lead to different TCR repertoires. Further studies with increased samples for sequencing or studies on structure analysis are warranted to reveal the underlying mechanisms.

In consideration of the currently-established herd immunity against SARS-CoV-2, our study revealed the characteristics and molecular mechanisms of the reverse preexisting immunity to other coronaviruses in the COVID-19 convalescents. Within the diverse T cell epitopes, due to its large proteome, the nonconserved peptide plays a dominant role in the cross-T cell reactivity. Our study provides new insights into the understanding of herd immunity to SARS-CoV-2 and the design of peptide-based universal vaccines to specific evolutionary branch of coronaviruses.

## Materials and methods

### Study participants

We recruited 95 COVID-19 convalescents from Macheng, Hubei, China (infected by Wuhan-Hu-1, NCBI Reference Sequence: MN908947.3) in July 2020 and January 2021, and 45 from Shijiazhuang, Hebei, China (infected by hCoV-19/Hebei/IVDC-01-01/2021, GISAID Reference Sequence: EPI_ISL_796012) in July 2021. They had not been vaccinated with any COVID-19 vaccines. In addition, 28 healthy controls (HCs) who had neither been infected with SARS-CoV-2 nor vaccinated against SARS-CoV-2 were also recruited from Beijing. We collected the basic information of them using a questionnaire survey (Supplementary Table 4). Peripheral venous blood was collected to isolate peripheral blood mononuclear cells (PBMCs). These PBMCs were stored in a cell stock solution containing 90% fetal bovine serum (FBS) and 10% dimethylsulfoxide, and then kept in liquid nitrogen for future use. The informed consents were obtained from the participants and this study was approved by the National Institute for Viral Disease Control and Prevention Ethics Committee of the Chinese Center for Disease Control and Prevention.

### Design and prediction of SARS-CoV and SARS-CoV-2 peptides

We designed 15- to 18-mer peptides, overlapping by 10 amino acids, to span the S, M, and N proteins of SARS-CoV-2 (Wuhan-Hu-1, NCBI Reference Sequence: MN908947.3) and SARS-CoV (SARS coronavirus Tor2, NCBI Reference Sequence: AY274119.3). The overlapping peptides of SARS-CoV-2 were assigned to two- or three-dimensional matrix systems, in which each peptide was represented in 2 or 3 different peptide pools (Supplementary Table 5). For positive overlapping peptides, we predicted a total of 97 short peptides (8- to 11-mer) to identify SARS-CoV-2 CD8^+^ T cell epitopes. Among them, we predicted 75 short peptides (8- to 11-mer) using the NetMHCpan-4.0/4.1 and summarized 22 short peptides according to the published SARS-CoV epitopes using the Immune Epitope Database.

When predicting CD8^+^ T cell epitope with NetMHCpan-4.0/4.1, we selected several common HLA alleles and used %Rank <2 and Affinity <500 as the prediction criteria. Then, we referred to human HLA-restricted anchored residue preference for prediction. In the HLA-A2-restricted peptide motif, the second and ninth positions are usually aliphatic amino acids L/I/V/M or A/G/S/T; In the HLA-A24-restricted peptide motif, the second position is usually Y/F, and the ninth position is F or W/L/I/C; In HLA-A11-restricted peptide motif, the second position is usually S/T or L/I/V/M, and the last position is usually K; In HLA-A33-restricted peptide motif, the second position is usually Y/F/S/T or L/I/V/M, usually ending with R.

### Design and synthesis of peptide pools

Peptides with identical sequence between SARS-CoV-2 and SARS-CoV in the positive overlapping peptides were classified as conserved peptides (Conserved-pool), with those with mismatched residues were considered SARS-CoV-2-specific peptides (Mut_SARS-CoV-2_-pool) and SARS-CoV-specific peptides (Mut_SARS-CoV_-pool), in which we obtained cross-reactive overlapping peptides. Within the overlapping peptides which could induce cross-reactivity, we summarized the published epitopes according to the Immune Epitope Database and synthesized 20 pairs of short CD8^+^ T cell peptides, which were classified into S-Mut_SARS-CoV-2_-pool and S-Mut_SARS-CoV_-pool, respectively.

### Expansion of SARS-CoV-2-specific T cells in vitro

PBMCs were seeded in 24-well plates (3 ×10^6^ cells/well) and cultured in RPMI-1640 (Gibco, USA) containing 10% FBS at 37 °C with 5% CO_2_. They were incubated with the peptide pools (2 μM/mL single peptide) and the recombinant IL-7 (20 U/mL, Pepro Tech, USA). On day 3, the recombinant IL-2 (20 U/mL, Pepro Tech, USA) was added to PBMCs. The culture medium was half replaced every 3 days. On day 10, the cells were collected and tested for the presence of virus-specific T cells.

### Enzyme-linked immunospot (ELISpot) assay

IFN-γ-secreting T cells were detected with ELISpot assay kits (BD Corp, USA). The 96-well ELISpot plate membranes were pre-coated with diluted anti-IFN-γ coating antibody overnight at 4 °C. After washing twice, the plate membranes were blocked with RPMI-1640 containing 10% FBS for 2 h at room temperature. Then, PBMCs and the stimulating peptides were added and incubated for 18 h at 37 °C. The cells stimulated with the phorbol ester (PMA) and without stimulation were used as positive and negative controls, respectively. After incubation, the cells were removed, and the plates were successively incubated with biotinylated IFN-γ detection antibodies, enzyme conjugate streptavidin-HRP and chromogenic substrate. The results are expressed as spot-forming cells (SFCs) among PBMCs, which were counted using an ELISpot Reader System (CTL-Immunospot S5 Versa, CTL Corp, USA). If the negative control had less than 5 SFCs/10^5^ PBMCs, positive responses were defined as having at least 10 SFCs/10^5^ PBMCs; otherwise, positive responses were defined as having PBMCs at least twice that of the negative control.

### Intracellular cytokine staining

After stimulation, the cells were incubated for 10 h at 37 °C in the presence of GolgiPlug (BD Biosciences, USA). Then, Zombie Yellow fixable viability dye (BioLegend, USA) was added to exclude dead cells. After that, cells were surface-stained for 30 min at 4 °C with anti-CD3-FITC, anti-CD4-PerCP-Cy5 and anti-CD8-BV510 (BioLegend, USA). After fixation and permeabilization for 20 min using BD Cytofix/Cytoperm solution (BD Biosciences, USA), the cells were intracellularly stained with anti-IFN-γ-PE-Cy7, anti-TNF-α-PE and anti-IL2-APC (BioLegend, USA) for 30 min. Non-specific cytokine production was identified with a no-peptide control. All samples were analyzed with flow cytometry. The gating strategy is shown in the Supplementary Fig. 2a.

### In vitro refolding of proteins

The refolding buffer was prepared and pre-cooled at 4 °C. The light chain (β_2_m), peptides, and heavy chains of different HLA types were successively added into the refolding buffer. The ratio of light chain to heavy chain was 1:3. After 8–10 h, the sample was concentrated. After concentrating to about 1 mL, the sample was loaded onto the molecular sieve column (AKTA pure).

### Circular dichroism spectrometry

The thermal stability of the HLA/peptide complexes was evaluated through circular dichroism spectrometry. All the HLA/peptide complexes were renatured and purified in 20 mM Tris-HCl (pH 8.0) and 50 mM NaCl. After adjusting the concentration of the HLA/peptide complex to 0.2 mg/mL, its spectrum at 218 nm was determined using the Chirascan spectrometer (Applied Photophysics). The set temperature increased from 20 °C to 80 °C at a rate of 1 °C/min and with a temperature interval of 0.2 °C. The denaturation curve and midpoint melting temperature of the measured proteins were determined using Origin 9.1 (OriginLab).

### Preparation and staining of tetramers

As previously described,^49^ HLA-A*0201 tetramers were prepared with the SARS-CoV-2-specific peptide SARS-CoV-2-N_262-270_ (RTATKAYNV) and SARS-CoV-specific peptide SARS-CoV-N_262-270_ (RTATKQYNV), while HLA-B*4601 tetramers were prepared with the SARS-CoV-2-N_266-274_ (KAYNVTQAF) and SARS-CoV-N_266-274_ (KQYNVTQAF). The cultured PBMCs were collected, washed, and then stained with anti-CD3-FITC, anti-CD8-BV510 (BioLegend, USA) and tetramers at 4 °C for 30 min. The cells stained with an irrelevant tetramer were used as negative controls. After washing, the cells were resuspended and immediately analyzed by flow cytometry in the dark.

### Single-cell T cell receptor (TCR) sequencing

After staining, tetramers-specific CD8^+^ T cells were sorted in 96-well plates using an 85-µm nozzle and fluorescence-activated cell sorting. Subsequently, primers and enzymes were added for reverse transcription and PCR amplification. The fragments of TCR were amplified using the primers of TCR α and β chains by two rounds of PCR. The amplified target fragments were determined by DNA agarose gel electrophoresis and extracted by MiniBest Agarose Gel DNA extraction kit (TaKaRa, Japan). Finally, the cDNA fragments were cloned into T vector using PEASY-Blunt Zero Cloning Kit (TRANSGEN, Beijing, China) and then subjected to TCR sequencing.

### Transfection of 293T cells

TCR plasmids were constructed with pCDH-CMV-MCS-EF1-Puro vectors. The 293T cells were seeded into 6-well plates and transfected with target plasmids (CD3CD8 and TCR) when the cell confluence reached 80–90%. In detail, transfection reagents and plasmids were mixed and incubated for 20 min. After that, the mixture was added to 293T cells, and the plate was shaken gently to ensure full contact of the mixture with cells. After 4 h, the culture medium was replaced with DMEM (Gibco, USA) containing 2% FBS. After another 24 h, the cells were collected and incubated with anti-CD3-APC, anti-CD8-BV510, anti-TCR-PerCP-Cy5 (BioLegend, USA) and Tetramers-PE for 30 min. Finally, the cells were analyzed by flow cytometry. The gating strategy for flow cytometry analysis is shown in the Supplementary Fig. 2b. The flowchart for the entire article is provided in Supplementary Fig. 3.

### Statistical analysis

Statistical analyses were conducted with SPSS 24.0 and GraphPad Prism 8. Continuous variables were presented as median (interquartile range) and compared with Mann–Whitney U-test and Wilcoxon matched-pairs signed rank test as appropriate. Categorical variables were presented as proportion and analyzed with chi-square test or Fisher’s exact test as appropriate. Simple linear regression was employed to assess the impact of disease severity on immune indexes. Statistical significance was defined as follows: not significant, * *P* < 0.05, ** *P* < 0.01, and *** *P* < 0.001. All tests were two tailed.

### Supplementary information

The online version contains supplementary materials available on the Signal Transduction and Targeted Therapy website http://www.nature.com/sigtrans.

### Supplementary information

Supplementary Materials

## Data Availability

The raw data of this study are available from the corresponding author Jun Liu (liujun@ivdc.chinacdc.cn) upon request for scientific purposes.
