# Supplementary material for: Nonconserved epitopes dominate reverse preexisting T cell immunity in COVID-19 convalescents
Source: Signal Transduct Target Ther. 2024 Jun 12;9:160. doi: 10.1038/s41392-024-01876-3 (PMC11169541; doi:10.1038/s41392-024-01876-3)
Supplement: Supplementary file 1 — Supplementary Materials [file 41392_2024_1876_MOESM1_ESM.docx]

Supplementary Materials for

Nonconserved epitopes dominate reverse preexisting T cell immunity in COVID-19 convalescents

Xin Wang, Jie Zhang, Maoshun Liu, Yuanyuan Guo, Peipei Guo, Xiaonan Yang, Bingli Shang, Min Li, Jinmin Tian, Ting Zhang, Xi Wang, Ronghua Jin, Jikun Zhou^,^ , George F. Gao, Jun Liu

Correspondence to: Jun Liu ([liujun@ivdc.chinacdc.cn](mailto:liujun@ivdc.chinacdc.cn)), George F.Gao ([gaof@im.ac.cn](mailto:gaof@im.ac.cn)), Jikun Zhou (13933880581@163.com)

**This PDF file includes:**

Figures. S1 to S3

Tables S1 to S5


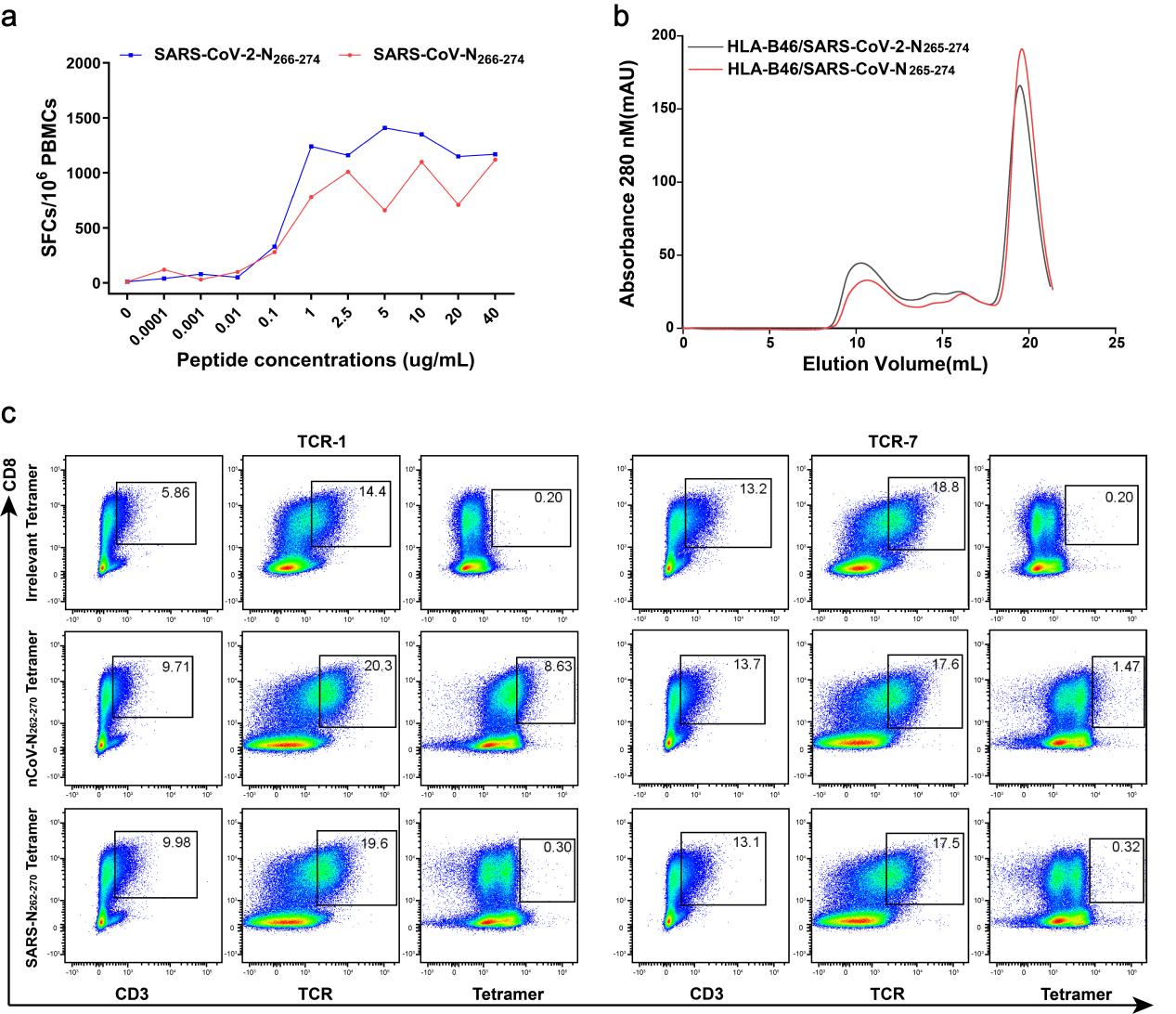


Figure. S1.

**(a)**, The peptide dose-response relationship of SARS-CoV-2-N_266-274_ and SARS-CoV-N_266-274_. (**b**), In vitro refolding of the SARS-CoV-2-N_265-274_ and SARS-CoV-N_265-274_ with HLA-B46. (**c**), The expresion of TCR-1 and -7 pairs and their binding to SARS-CoV-N_262-270_ and SARS-CoV-2-N_262-270_ tetramers.


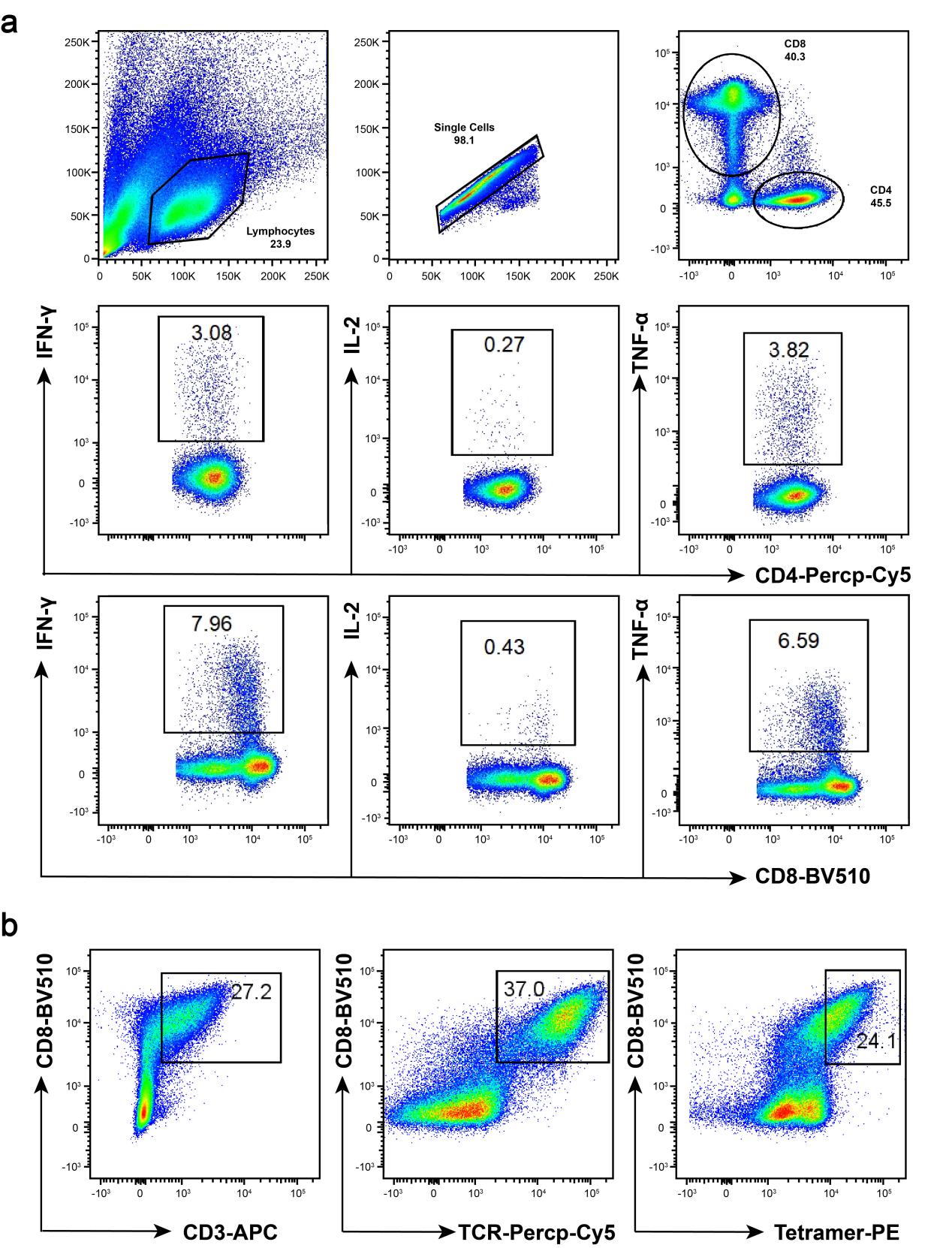


Figure. S2.

**The gating strategy for flow cytometry analysis. (a)**, The gating strategy of intracellular cytokine staining. **(b)**, The gating strategy of Transfection of 293T cells.


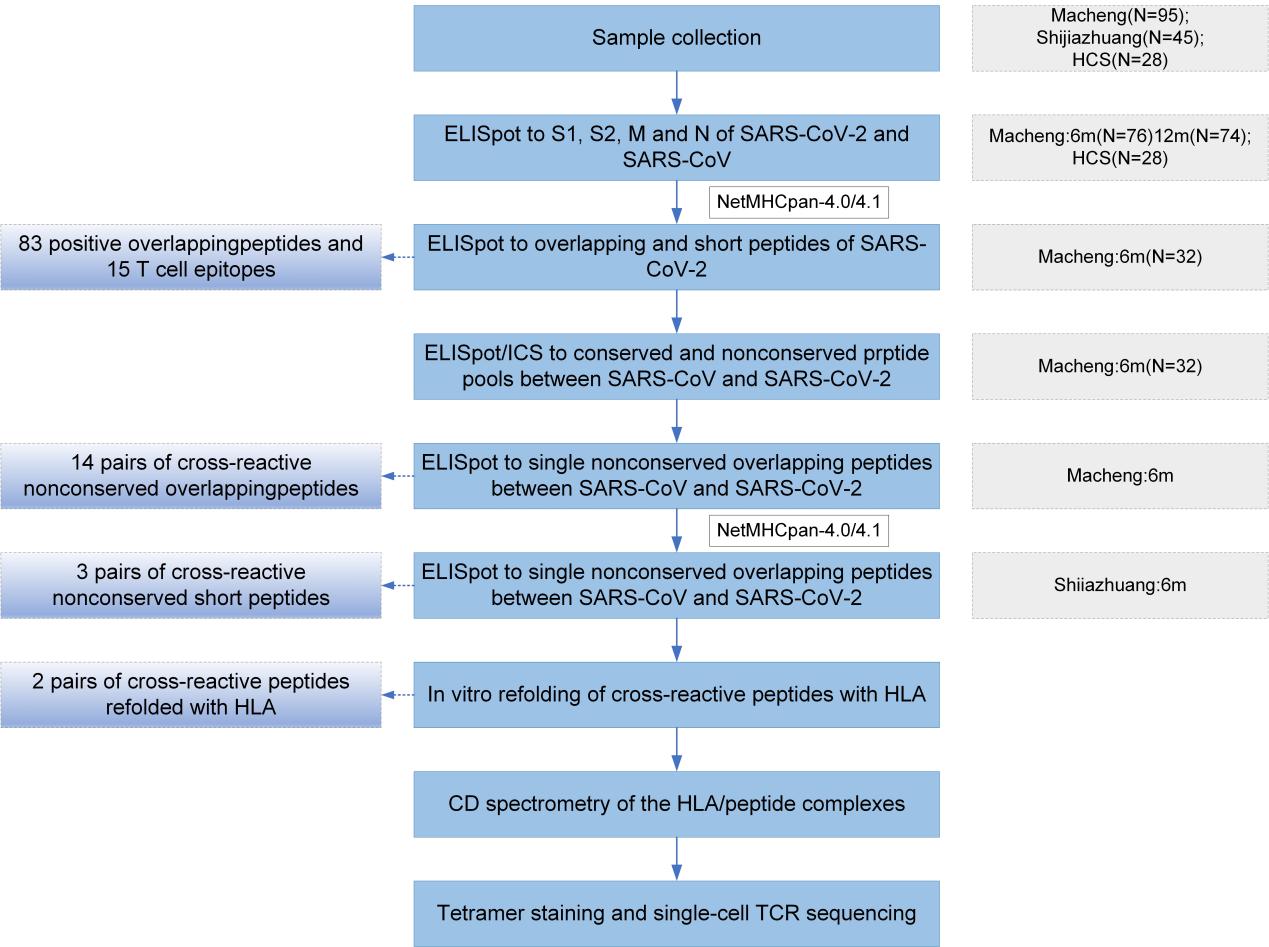


Figure. S3.

The flowchart for the entire study.

**Table S1.**

**A total of 83 positive overlapping peptides, including 53 from the S protein (31 from S1 and 22 from S2), 12 from the M protein, and 18 from the N protein.**

| **Name** | **Position** | **SARS-CoV-2 amino acid sequence** | **SARS-CoV amino acid sequence** |
| --- | --- | --- | --- |
| S2 | 8-24 | LPLVSSQCVNLTTRTQL | GSDLDRCTTFDDVQAPN |
| S5 | 24-41 | LPPAYTNSFTRGVYYPDK | PNYTQHTSSMRGVYYPDE |
| S6 | 31-48 | SFTRGVYYPDKVFRSSVL | SSMRGVYYPDEIFRSDTL |
| S7 | 38-55 | YPDKVFRSSVLHSTQDLF | YPDEIFRSDTLYLTQDLF |
| S10 | 59-76 | FSNVTWFHAIHVSGTNGT | YSNVTGFHTINHTFDN |
| S12 | 73-90 | TNGTKRFDNPVLPFNDGV | HTINHTFDNPVIPFKDGI |
| S14 | 87-104 | NDGVYFASTEKSNIIRGW | KDGIYFAATEKSNVVRGW |
| S15 | 94-110 | STEKSNIIRGWIFGTTL | ATEKSNVVRGWVFGSTM |
| S16 | 100-117 | IIRGWIFGTTLDSKTQSL | VVRGWVFGSTMNNKSQSV |
| S21 | 131-147 | CEFQFCNDPFLGVYYHK | CNFELCDNPFFAVSKPM |
| S26 | 161-177 | SSANNCTFEYVSQPFLM | DNAFNCTFEYISDAFSL |
| S27 | 167-182 | TFEYVSQPFLMDLEGK | TFEYISDAFSLDVSEK |
| S31 | 192-207 | FVFKNIDGYFKIYSKH | FVFKNKDGFLYVYKGY |
| S32 | 197-214 | IDGYFKIYSKHTPINLVR | KDGFLYVYKGYQPIDVVR |
| S33 | 204-220 | YSKHTPINLVRDLPQGF | YKGYQPIDVVRDLPSGF |
| S34 | 210-227 | INLVRDLPQGFSALEPLV | IDVVRDLPSGFNTLKPIF |
| S42 | 260-277 | AGAAAYYVGYLQPRTFLL | TSAAAYFVGYLKPTTFML |
| S43 | 267-284 | VGYLQPRTFLLKYNENGT | VGYLKPTTFMLKYDENGT |
| S55 | 345-362 | TRFASVYAWNRKRISNCV | TKFPSVYAWERKKISNCV |
| S56 | 352-369 | AWNRKRISNCVADYSVLY | AWERKKISNCVADYSVLY |
| S61 | 382-397 | VSPTKLNDLCFTNVYA | VSATKLNDLCFSNVYA |
| S63 | 393-410 | TNVYADSFVIRGDEVRQI | SNVYADSFVVKGDDVRQI |
| S64 | 400-417 | FVIRGDEVRQIAPGQTGK | FVVKGDDVRQIAPGQTGV |
| S71 | 442-458 | DSKVGGNYNYLYRLFRK | DATSTGNYNYKYRYLRH |
| S72 | 448-464 | NYNYLYRLFRKSNLKPF | NYNYKYRYLRHGKLRPF |
| S81 | 505-522 | YQPYRVVVLSFELLHAPA | YQPYRVVVLSFELLNAPA |
| S82 | 512-529 | VLSFELLHAPATVCGPKK | VLSFELLNAPATVCGPKL |
| S84 | 525-541 | CGPKKSTNLVKNKCVNF | CGPKLSTDLIKNQCVNF |
| S89 | 555-570 | SNKKFLPFQQFGRDIA | SSKRFQPFQQFGRDVS |
| S91 | 567-584 | RDIADTTDAVRDPQTLEI | RDVSDFTDSVRDPKTSEI |
| S95 | 595-612 | VSVITPGTNTSNQVAVLY | VSVITPGTNASSEVAVLY |
| S108 | 682-699 | RRARSVASQSIIAYTMSL | SLLRSTSQKSIVAYTMSL |
| S118 | 749-766 | CSNLLLQYGSFCTQLNRA | CANLLLQYGSFCTQLNRA |
| S123 | 780-797 | EVFAQVKQIYKTPPIKDF | EVFAQVKQMYKTPTLKYF |
| S124 | 787-802 | QIYKTPPIKDFGGFNF | QMYKTPTLKYFGGFNF |
| S127 | 801-818 | NFSQILPDPSKPSKRSFI | NFSQILPDPLKPTKRSFI |
| S129 | 815-831 | RSFIEDLLFNKVTLADA |  |
| S137 | 861-878 | LPPLLTDEMIAQYTSALL | LPPLLTDDMIAAYTAALV |
| S138 | 868-882 | EMIAQYTSALLAGTI | DMIAAYTAALVSGTA |
| S143 | 896-911 | IPFAMQMAYRFNGIGV |  |
| S145 | 907-924 | NGIGVTQNVLYENQKLIA | NGIGVTQNVLYENQKQIA |
| S147 | 921-938 | KLIANQFNSAIGKIQDSL | KQIANQFNKAISQIQESL |
| S152 | 956-973 | AQALNTLVKQLSSNFGAI |  |
| S157 | 990-1007 | EVQIDRLITGRLQSLQTY |  |
| S158 | 997-1004 | ITGRLQSLQTYVTQQLIR |  |
| S159 | 1004-1020 | LQTYVTQQLIRAAEIRA |  |
| S161 | 1016-1033 | AEIRASANLAATKMSEYV | AEIRASANLAATKMSECV |
| S165 | 1042-1058 | FCGKGYHLMSFPQSAPH | FCGKGYHLMSFPQAAPH |
| S166 | 1048-1065 | HLMSFPQSAPHGVVFLHV | HLMSFPQAAPHGVVFLHV |
| S167 | 1055-1070 | SAPHGVVFLHVTYVPA | AAPHGVVFLHVTYVPS |
| S168 | 1060-1075 | VVFLHVTYVPAQEKNF | VVFLHVTYVPSQERNF |
| S174 | 1099-1115 | GTHWFVTQRNFYEPQII | GTSWFITQRNFFSPQII |
| S179 | 1128-1145 | VVIGIVNNTVYDPLQPEL | VVIGIINNTVYDPLQPEL |
| M12 | 83-100 | AMACLVGLMWLSYFIASF | AMACIVGLMWLSYFVASF |
| M14 | 98-112 | ASFRLFARTRSMWSF |  |
| M15 | 103-120 | FARTRSMWSFNPETNILL |  |
| M16 | 111-128 | SFNPETNILLNVPLHGTI | SFNPETNILLNVPLRGTI |
| M20 | 141-158 | GAVILRGHLRIAGHHLGR | GAVIIRGHLRMAGHSLGR |
| M21 | 149-166 | LRIAGHHLGRCDIKDLPK | LRMAGHSLGRCDIKDLPK |
| M22 | 157-174 | GRCDIKDLPKEITVATSR |  |
| M23 | 165-181 | PKEITVATSRTLSYYKL |  |
| M24 | 172-188 | TSRTLSYYKLGASQRVA | TSRTLSYYKLGASQRV |
| M25 | 179-196 | YKLGASQRVAGDSGFAAY | YKLGASQRVGDSGFAA |
| M26 | 187-204 | VAGDSGFAAYSRYRIGNY | RVGDSGFAAYNRYRIGNY |
| M27 | 195-210 | AYSRYRIGNYKLNTDH | AYNRYRIGNYKLNTDH |
| N1 | 1-17 | MSDNGPQNQRNAPRITF | MSDNGPQSNQRSAPRITF |
| N8 | 52-68 | WFTALTQHGKEDLKFPR | WFTALTQHGKEELRFPR |
| N12 | 81-95 | DDQIGYYRRATRRIR | DDQIGYYRRATRRVR |
| N13 | 86-102 | YYRRATRRIRGGDGKMK | YYRRATRRVRGGDGKMK |
| N14 | 93-110 | RIRGGDGKMKDLSPRWYF | RVRGGDGKMKELSPRWYF |
| N15 | 101-118 | MKDLSPRWYFYYLGTGPE | MKELSPRWYFYYLGTGPE |
| N16 | 109-125 | YFYYLGTGPEAGLPYGA | YFYYLGTGPEASLPYGA |
| N18 | 124-139 | GANKDGIIWVATEGAL | GANKEGIVWVATEGAL |
| N19 | 130-146 | IIWVATEGALNTPKDHI | IVWVATEGALNTPKDHI |
| N28 | 194-211 | SRNSTPGSSRGTSPARMA | SRNSTPGSSRGNSPARMA |
| N30 | 210-227 | MAGNGGDAALALLLLDRL | MASGGGETALALLLLDRL |
| N36 | 253-270 | EASKKPRQKRTATKAYNV | EASKKPRQKRTATKQYNV |
| N37 | 261-277 | KRTATKAYNVTQAFGRR | KRTATKQYNVTQAFGRR |
| N43 | 305-322 | AQFAPSASAFFGMSRIGM |  |
| N44 | 313-330 | AFFGMSRIGMEVTPSGTW |  |
| N45 | 321-338 | GMEVTPSGTWLTYTGAIK | GMEVTPSGTWLTYHGAIK |
| N46 | 329-346 | TWLTYTGAIKLDDKDPNF | TWLTYHGAIKLDDKDPQF |
| N48 | 344-361 | PNFKDQVILLNKHIDAYK | PQFKDNVILLNKHIDAYK |

**Table S2.**

**A total of 97 short peptides (8- to 11-mer) within the 83 positive overlapping peptides predicted with NetMHCpan and IEDB.**

| **Position** | **SARS-CoV-2 amino acid sequence** | **Predicted HLA restriction** |
| --- | --- | --- |
| 78-86 | RFDNPVLPF | HLA-A2403 |
| 170-179 | YVSQPFLMDL | HLA-A0217 |
| 168-175 | FEYVSQPF | HLA-B1801 |
| 168-176 | FEYVSQPFL | HLA-B4001 |
| 192-200 | FVFKNIDGY | HLA-B1502 |
| 202-210 | KIYSKHTPI | HLA-A0203 |
| 448-456 | NYNYLYRLF | HLA-A2403 |
| 456-464 | FRKSNLKPF | HLA-B1503 |
| 453-462 | YRLFRKSNLK | HLA-B2705 |
| 453-461 | YRLFRKSNL | HLA-B2720 |
| 1062-1070 | FLHVTYVPA | HLA-A0203 |
| 1060-1068 | VVFLHVTYV | HLA-A0206 |
| 28-36 | YTNSFTRGV | HLA-A0203 |
| 100-108 | IIRGWIFGT | HLA-A0211 |
| 109-117 | TLDSKTQSL | HLA-A0219 |
| 507-515 | PYRVVVLSF | HLA-A2403 |
| 869-877 | MIAQYTSAL | HLA-A0202 |
| 1101-1109 | HWFVTQRNF | HLA-A2403 |
| 108-116 | SMWSFNPET | HLA-A0201 |
| 112-120 | FNPETNILL | HLA-A0217 |
| 103-112 | FARTRSMWSF | HLA-B2705 |
| 148-156 | HLRIAGHHL | HLA-A0250 |
| 171-180 | ATSRTLSYYK | HLA-A1101 |
| 169-178 | TVATSRTLSY | HLA-B1501 |
| 171-179 | ATSRTLSYY | HLA-B1517 |
| 168-176 | ITVATSRTL | HLA-B1517 |
| 170-178 | VATSRTLSY | HLA-B1517 |
| 86-94 | YYRRATRRI | HLA-A2301 |
| 266-274 | KAYNVTQAF | HLA-B1501 |
| 267-274 | AYNVTQAF | HLA-A2403 |
| 315-324 | FGMSRIGMEV | HLA-A0202 |
| 316-324 | GMSRIGMEV | HLA-A0202 |
| 329-337 | TWLTYTGAI | HLA-A2403 |
| 351-359 | ILLNKHIDA | HLA-A0216 |
| 352-360 | LLNKHIDAY | HLA-B1501 |
| 38-47 | YPDKVFRSSV | HLA-A0207 |
| 89-97 | GVYFASTEK | HLA-A1101 |
| 100-108 | IIRGWIFGT | HLA-A0211 |
| 131-140 | CEFQFCNDPF | HLA-A2301 |
| 268-277 | GYLQPRTFLL | HLA-A2301 |
| 264-272 | AYYVGYLQP | HLA-A2403 |
| 268-276 | GYLQPRTFL | HLA-A2403 |
| 269-278 | YLQPRTFLLK | HLA-A1101 |
| 348-356 | ASVYAWNRK | HLA-A1101 |
| 444-452 | KVGGNYNYL | HLA-A2403 |
| 915-923 | VLYENQKLI | HLA-A0212 |
| 691-699 | SIIAYTMSL | HLA-A0201 |
| 687-695 | VASQSIIAY | HLA-B1503 |
| 684-692 | ARSVASQSI | HLA-B2720 |
| 689-697 | SQSIIAYTM | HLA-B3901 |
| 754-763 | LQYGSFCTQL | HLA-A0206 |
| 753-761 | LLQYGSFCT | HLA-A0212 |
| 755-763 | QYGSFCTQL | HLA-A2403 |
| 786-794 | KQIYKTPPI | HLA-A0206 |
| 821-829 | LLFNKVTLA | HLA-A0201 |
| 817-825 | FIEDLLFNK | HLA-A1101 |
| 869-877 | MIAQYTSAL | HLA-A0202 |
| 901-909 | QMAYRFNGI | HLA-A0203 |
| 901-909 | QMAYRFNGI | HLA-A0212 |
| 915-923 | VLYENQKLI | HLA-A0212 |
| 965-973 | QLSSNFGAI | HLA-A0205 |
| 962-970 | LVKQLSSNF | HLA-B1501 |
| 1000-1008 | RLQSLQTYV | HLA-A0201 |
| 1005-1013 | QTYVTQQLI | HLA-B1517 |
| 1032-1040 | YVLGQSKRV | HLA-A0219 |
| 1050-1058 | MSFPQSAPH | HLA-B1517 |
| 1054-1062 | QSAPHGVVF | HLA-B1517 |
| 1121-1129 | FVSGNCDVV | HLA-A0202 |
| 93-101 | LSYFIASFR | HLA-A1101 |
| 94-102 | SYFIASFRL | HLA-A2301 |
| 95-103 | YFIASFRLF | HLA-A2301 |
| 119-127 | LLNVPLHGT | HLA-A0250 |
| 114-123 | GTGPEAGLPY | HLA-B1501 |
| 259-268 | RQKRTATKAY | HLA-B1501 |
| 306-314 | QFAPSASAF | HLA-A2403 |
| S411-420 | KLPDDFTGCV | HLA-A*0201 |
| S787-795 | ILPDPSKPS | HLA-A*0201 |
| S1042-1050 | VVFLHVTYV | HLA-A2 |
| S958-966 | VLNDILSRL | HLA-A*0201 |
| S978-986 | LITGRLQSL | HLA-A2 |
| S1203-1211 | FIAGLIAIV | HLA-A2 |
| S1167-1175 | RLNEVAKNL | HLA-A*0201 |
| S1174-1182 | NLNESLIDL | HLA-A*0201 |
| N216-224 | GDAALALLL | HLA-B*4001 |
| N323-332 | MEVTPSGTWL | HLA-B*4001 |
| N223-231 | LLLDRLNQL | HLA-A*0201 |
| N227-235 | RLNQLESKM | HLA-A*0201 |
| N317-325 | GMSRIGMEV | HLA-A*0201 |
| N220-228 | LALLLLDRL | HLA-A*0201 |
| N216-225 | GDAALALLLL | HLA-B*4001 |
| N222-231 | LLLLDRLNQL | HLA-A*0201 |
| N266–275 | TKAYNVTQAF | HLA-B*1525 |
| N346-354 | NFKDQVILL | HLA-A*2402 |
| N362-370 | KTFPPTEPK | HLA-A*1101 |
| M60-69 | TLACFVLAAV | HLA-A*0201 |
| M88-96 | GLMWLSYFI | HLA-A*0201 |
| M147-155 | HLRIAGHHL | HLA-B*1502 |

**Table S3.**

**The sequences of TCR α chain and β chain for the SARS-CoV-2-N_262-270_ and SARS-CoV-N_262-270_ peptides.**

|  | **α chain** | | | **β chain** | | | |
| --- | --- | --- | --- | --- | --- | --- | --- |
|  | **TRAV** | **TRAJ** | **CDR3α** | **TRBV** | **TRBJ** | **TRBD** | **CDR3β** |
| SARS-CoV-2 | Homsap TRAV13-2*01 F | Homsap TRAJ15*01 F | CAALNQAGTALIF | Homsap TRBV20-1*05 (F) | Homsap TRBJ1-6*01 F | Homsap TRBD2*01 F | CSASHPGDSPLHF |
|  | Homsap TRAV17*01 F | Homsap TRAJ47*01 F | CATDEEEYGNKLVF | Homsap TRBV15*02 F | Homsap TRBJ1-2*01 F | Homsap TRBD2*01 F | CATSDYNRDFYGYTF |
|  | Homsap TRAV5*01 F | Homsap TRAJ42*01 F | CAESIGYGGSQGNLIF | Homsap TRBV11-2*01 F | Homsap TRBJ2-3*01 F | Homsap TRBD2*01 F | CASSLGPGLASDTQYF |
|  | Homsap TRAV17*01 F | Homsap TRAJ47*01 F | CATDEEEYGNKLVF | Homsap TRBV2*01 F | Homsap TRBJ1-4*01 F | Homsap TRBD1*01 F | CASSDYNAEKLFF |
|  | Homsap TRAV17*01 F | Homsap TRAJ47*01 F | CATDEEEYGNKLVF | Homsap TRBV20-1*05 (F) | Homsap TRBJ1-6*01 F | Homsap TRBD2*01 F | CSASHPGDSPLHF |
|  | Homsap TRAV17*01 F | Homsap TRAJ47*01 F | CATDEEEYGNKLVF | Homsap TRBV11-2*01 F | Homsap TRBJ2-3*01 F | Homsap TRBD2*01 F | CASSLGPGLASDTQYF |
|  | Homsap TRAV17*01 F | Homsap TRAJ27*01 F | CATDEDTNAGKSTF | Homsap TRBV2*01 F | Homsap TRBJ1-4*01 F | Homsap TRBD1*01 F | CASSDYNAEKLFF |
|  | Homsap TRAV4*01 F | Homsap TRAJ34*01 F | CLVGDVDTDKLIF | Homsap TRBV11-2*01 F | Homsap TRBJ2-3*01 F | Homsap TRBD2*01 F | CASSLGPGLASDTQYF |
|  | Homsap TRAV17*01 F | Homsap TRAJ47*01 F | CATDEEEYGNKLVF | Homsap TRBV2*01 F | Homsap TRBJ1-4*01 F | Homsap TRBD1*01 F | CASSDYNAEKLFF |
|  | Homsap TRAV4*01 F | Homsap TRAJ34*01 F | CLVGDVDTDKLIF | Homsap TRBV13*01 F | Homsap TRBJ2-5*01 F |  | CASSLQNQETQYF |
|  | Homsap TRAV17*01 F | Homsap TRAJ47*01 F | CATDEEEYGNKLVF | Homsap TRBV2*01 F | Homsap TRBJ1-4*01 F | Homsap TRBD1*01 F | CASSDYNAEKLFF |
|  | Homsap TRAV17*01 F | Homsap TRAJ47*01 F | CATDEEEYGNKLVF | Homsap TRBV2*01 F | Homsap TRBJ1-4*01 F | Homsap TRBD1*01 F | CASSDYNAEKLFF |
|  | Homsap TRAV17*01 F | Homsap TRAJ47*01 F | CATDEEEYGNKLVF | Homsap TRBV20-1*05 (F) | Homsap TRBJ1-6*01 F | Homsap TRBD2*01 F | CSASHPGDSPLHF |
|  | Homsap TRAV4*01 F | Homsap TRAJ34*01 F | CLVGDVDTDKLIF | Homsap TRBV20-1*05 (F) | Homsap TRBJ1-6*01 F | Homsap TRBD2*01 F | CSASHPGDSPLHF |
|  | Homsap TRAV17*01 F | Homsap TRAJ47*01 F | CATDEEEYGNKLVF | Homsap TRBV20-1*05 (F) | Homsap TRBJ1-6*01 F | Homsap TRBD2*01 F | CSASHPGDSPLHF |
|  | Homsap TRAV17*01 F | Homsap TRAJ27*01 F | CATDEDTNAGKSTF | Homsap TRBV2*01 F | Homsap TRBJ1-4*01 F | Homsap TRBD1*01 F | CASSDYNAEKLFF |
|  | Homsap TRAV4*01 F | Homsap TRAJ34*01 F | CLVGDVDTDKLIF | Homsap TRBV2*01 F | Homsap TRBJ1-4*01 F | Homsap TRBD1*01 F | CASSDYNAEKLFF |
|  | Homsap TRAV17*01 F | Homsap TRAJ47*01 F | CATDEEEYGNKLVF | Homsap TRBV11-2*01 F | Homsap TRBJ2-3*01 F | Homsap TRBD2*01 F | CASSLGPGLASDTQYF |
|  | Homsap TRAV5*01 F | Homsap TRAJ42*01 F | CAESIGYGGSQGNLIF | Homsap TRBV2*01 F | Homsap TRBJ1-4*01 F | Homsap TRBD1*01 F | CASSDYNAEKLFF |
|  | Homsap TRAV4*01 F | Homsap TRAJ34*01 F | CLVVLEEDTDKLIF | Homsap TRBV13*01 F | Homsap TRBJ2-5*01 F |  | CASSLQNQETQYF |
|  | Homsap TRAV17*01 F | Homsap TRAJ27*01 F | CATDEDTNAGKSTF | Homsap TRBV27*01 F | Homsap TRBJ1-1*01 F | Homsap TRBD1*01 F | CASSLSPAGSDSEAFF |
|  | Homsap TRAV17*01 F | Homsap TRAJ21*01 F | CATDADNFNKFYF | Homsap TRBV13*01 F | Homsap TRBJ2-5*01 F |  | CASSLQNQETQYF |
|  | Homsap TRAV17*01 F | Homsap TRAJ47*01 F | CATDEEEYGNKLVF | Homsap TRBV2*01 F | Homsap TRBJ1-4*01 F | Homsap TRBD1*01 F | CASSDYNAEKLFF |
|  | Homsap TRAV17*01 F | Homsap TRAJ47*01 F | CATDEEEYGNKLVF | Homsap TRBV11-2*01 F | Homsap TRBJ2-3*01 F | Homsap TRBD2*01 F | CASSLGPGLASDTQYF |
|  | Homsap TRAV5*01 F | Homsap TRAJ42*01 F | CAESIGYGGSQGNLIF | Homsap TRBV10-3*02 F | Homsap TRBJ1-6*01 F | Homsap TRBD1*01 F | CAISESNGALHSPLHF |
|  | Homsap TRAV4*01 F | Homsap TRAJ34*01 F | CLVGDVDTDKLIF | Homsap TRBV4-1*01 F | Homsap TRBJ1-1*01 F | Homsap TRBD2*01 F | CASSPDPWGVDTEAFF |
|  | Homsap TRAV17*01 F | Homsap TRAJ47*01 F | CATDEEEYGNKLVF | Homsap TRBV28*01 F | Homsap TRBJ2-1*01 F | Homsap TRBD1*01 F | CASTLRGVREQFF |
|  | Homsap TRAV4*01 F | Homsap TRAJ34*01 F | CLVGDVDTDKLIF | Homsap TRBV2*01 F | Homsap TRBJ1-4*01 F | Homsap TRBD1*01 F | CASSDYNAEKLFF |
|  | Homsap TRAV17*01 F | Homsap TRAJ47*01 F | CATDEEEYGNKLVF | Homsap TRBV13*01 F | Homsap TRBJ2-5*01 F | Homsap TRBD2*01 F | CASSSRLAGQETQYF |
|  | Homsap TRAV17*01 F | Homsap TRAJ47*01 F | CATDEEEYGNKLVF | Homsap TRBV2*01 F | Homsap TRBJ1-4*01 F | Homsap TRBD1*01 F | CASSDYNAEKLFF |
|  | Homsap TRAV17*01 F | Homsap TRAJ47*01 F | CATDEEEYGNKLVF | Homsap TRBV20-1*05 (F) | Homsap TRBJ1-6*01 F | Homsap TRBD2*01 F | CSASHPGDSPLHF |
|  | Homsap TRAV17*01 F | Homsap TRAJ47*01 F | CATDEEEYGNKLVF | Homsap TRBV20-1*05 (F) | Homsap TRBJ1-6*01 F | Homsap TRBD2*01 F | CSASHPGDSPLHF |
|  | Homsap TRAV5*01 F | Homsap TRAJ42*01 F | CAESIGYGGSQGNLIF | Homsap TRBV2*01 F | Homsap TRBJ1-4*01 F | Homsap TRBD1*01 F | CASSDYNAEKLFF |
|  | Homsap TRAV17*01 F | Homsap TRAJ47*01 F | CATDEEEYGNKLVF | Homsap TRBV2*01 F | Homsap TRBJ1-4*01 F | Homsap TRBD1*01 F | CASSDYNAEKLFF |
|  | Homsap TRAV29/DV5*01 F | Homsap TRAJ47*01 F | CAPTNGNKLVF | Homsap TRBV20-1*05 (F) | Homsap TRBJ1-6*01 F | Homsap TRBD2*01 F | CSASHPGDSPLHF |
|  | Homsap TRAV1-2*01 F | Homsap TRAJ21*01 F | CAVSNFNKFYF | Homsap TRBV13*01 F | Homsap TRBJ2-5*01 F | Homsap TRBD2*01 F | CASSSRLAGQETQYF |
|  | Homsap TRAV17*01 F | Homsap TRAJ47*01 F | CATDEEEYGNKLVF | Homsap TRBV2*01 F | Homsap TRBJ1-4*01 F | Homsap TRBD1*01 F | CASSDYNAEKLFF |
|  | Homsap TRAV17*01 F | Homsap TRAJ47*01 F | CATDEEEYGNKLVF | Homsap TRBV2*01 F | Homsap TRBJ1-4*01 F | Homsap TRBD1*01 F | CASSDYNAEKLFF |
|  | Homsap TRAV17*01 F | Homsap TRAJ47*01 F | CATDEEEYGNKLVF | Homsap TRBV13*01 F | Homsap TRBJ2-5*01 F |  | CASSLQNQETQYF |
|  | Homsap TRAV17*01 F | Homsap TRAJ47*01 F | CATDEEEYGNKLVF | Homsap TRBV7-9*03 F | Homsap TRBJ2-7*01 F | Homsap TRBD1*01 F | CASSPGVQYEQYF |
|  | Homsap TRAV17*01 F | Homsap TRAJ47*01 F | CATDEEEYGNKLVF | Homsap TRBV3-1*01 F | Homsap TRBJ2-7*01 F | Homsap TRBD2*01 F | CASRGERGYEQYF |
|  | Homsap TRAV38-2/DV8*01 F | Homsap TRAJ42*01 F | CAYSYGGSQGNLIF | Homsap TRBV6-4*01 F | Homsap TRBJ2-3*01 F | Homsap TRBD2*02 F | CASSESGRATDTQYF |
|  | Homsap TRAV17*01 F | Homsap TRAJ47*01 F | CATDEEEYGNKLVF | Homsap TRBV27*01 F | Homsap TRBJ1-1*01 F | Homsap TRBD1*01 F | CASSLSPAGSDSEAFF |
|  | Homsap TRAV4*01 F | Homsap TRAJ34*01 F | CLVVLEEDTDKLIF | Homsap TRBV2*01 F | Homsap TRBJ1-4*01 F | Homsap TRBD1*01 F | CASSDYNAEKLFF |
|  | Homsap TRAV17*01 F | Homsap TRAJ47*01 F | CATDEEEYGNKLVF | Homsap TRBV13*01 F | Homsap TRBJ2-7*02 ORF | Homsap TRBD1*01 F | CASSSLTGGDEQYV |
|  | Homsap TRAV17*01 F | Homsap TRAJ47*01 F | CATDEEEYGNKLVF | Homsap TRBV2*01 F | Homsap TRBJ1-4*01 F | Homsap TRBD1*01 F | CASSDYNAEKLFF |
|  | Homsap TRAV38-1*03 (F) | Homsap TRAJ57*01 F | CAFIVPQGGSEKLVF | Homsap TRBV27*01 F | Homsap TRBJ1-1*01 F | Homsap TRBD1*01 F | CASSLSPAGSDSEAFF |
|  | Homsap TRAV17*01 F | Homsap TRAJ27*01 F | CATDEDTNAGKSTF | Homsap TRBV4-1*01 F | Homsap TRBJ1-1*01 F | Homsap TRBD2*01 F | CASSPDPWGVDTEAFF |
|  | Homsap TRAV12-1*01 F | Homsap TRAJ12*01 F | CVVLMDSSYKLIF | Homsap TRBV2*01 F | Homsap TRBJ1-4*01 F | Homsap TRBD1*01 F | CASSDYNAEKLFF |
|  | Homsap TRAV12-1*01 F | Homsap TRAJ12*01 F | CVVLMDSSYKLIF | Homsap TRBV29-1*01 F | Homsap TRBJ1-2*01 F | Homsap TRBD1*01 F | CSVVPTGGYGYTF |
|  | Homsap TRAV1-1*01 F | Homsap TRAJ33*01 F | CAPRGSCVDSNYQLIW | Homsap TRBV15*02 F | Homsap TRBJ1-2*01 F | Homsap TRBD2*01 F | CATSDYNRDFYGYTF |
|  | Homsap TRAV17*01 F | Homsap TRAJ47*01 F | CATDEEEYGNKLVF | Homsap TRBV27*01 F | Homsap TRBJ1-1*01 F | Homsap TRBD1*01 F | CASSLSPAGSDSEAFF |
|  | Homsap TRAV17*01 F | Homsap TRAJ47*01 F | CATDEEEYGNKLVF | Homsap TRBV4-1*01 F | Homsap TRBJ1-1*01 F | Homsap TRBD2*01 F | CASSPDPWGVDTEAFF |
|  | Homsap TRAV17*01 F | Homsap TRAJ47*01 F | CATDEEEYGNKLVF | Homsap TRBV27*01 F | Homsap TRBJ1-1*01 F | Homsap TRBD1*01 F | CASSLSPAGSDSEAFF |
|  | Homsap TRAV26-2*01 F | Homsap TRAJ58*01 ORF | CILRPQEETSGSRLTF | Homsap TRBV27*01 F | Homsap TRBJ1-1*01 F | Homsap TRBD1*01 F | CASSLSPAGSDSEAFF |
|  | Homsap TRAV4*01 F | Homsap TRAJ34*01 F | CLVGDVDTDKLIF | Homsap TRBV13*01 F | Homsap TRBJ2-7*02 ORF | Homsap TRBD1*01 F | CASSSLTGGDEQYV |
|  | Homsap TRAV4*01 F | Homsap TRAJ34*01 F | CLVGDVDTDKLIF | Homsap TRBV2*01 F | Homsap TRBJ1-4*01 F | Homsap TRBD1*01 F | CASSDYNAEKLFF |
|  | Homsap TRAV17*01 F | Homsap TRAJ47*01 F | CATDEEEYGNKLVF | Homsap TRBV2*01 F | Homsap TRBJ1-4*01 F | Homsap TRBD1*01 F | CASSDYNAEKLFF |
|  | Homsap TRAV17*01 F | Homsap TRAJ47*01 F | CATDEEEYGNKLVF | Homsap TRBV2*01 F | Homsap TRBJ1-4*01 F | Homsap TRBD1*01 F | CASSDYNAEKLFF |
|  | Homsap TRAV12-1*01 F | Homsap TRAJ12*01 F | CVVLMDSSYKLIF | Homsap TRBV13*01 F | Homsap TRBJ1-1*01 F | Homsap TRBD1*01 F | CASSKGVKENTEAFF |
|  | Homsap TRAV4*01 F | Homsap TRAJ34*01 F | CLVGDVDTDKLIF | Homsap TRBV6-5*01 F | Homsap TRBJ2-5*01 F | Homsap TRBD2*01 F | CASSFLIAGRETQYF |
|  | Homsap TRAV17*01 F | Homsap TRAJ47*01 F | CATDEEEYGNKLVF | Homsap TRBV20-1*05 (F) | Homsap TRBJ1-6*01 F | Homsap TRBD2*01 F | CSASHPGDSPLHF |
|  | Homsap TRAV38-2/DV8*01 F | Homsap TRAJ42*01 F | CAYSYGGSQGNLIF | Homsap TRBV20-1*05 (F) | Homsap TRBJ1-6*01 F | Homsap TRBD2*01 F | CSASHPGDSPLHF |
|  | Homsap TRAV12-1*01 F | Homsap TRAJ12*01 F | CVVEMDSSYKLIF | Homsap TRBV27*01 F | Homsap TRBJ1-1*01 F | Homsap TRBD1*01 F | CASSLSPAGSDSEAFF |
|  | Homsap TRAV38-2/DV8*01 F | Homsap TRAJ42*01 F | CAYSYGGSQGNLIF | Homsap TRBV2*01 F | Homsap TRBJ1-4*01 F | Homsap TRBD1*01 F | CASSDYNAEKLFF |
|  | Homsap TRAV17*01 F | Homsap TRAJ47*01 F | CATDEEEYGNKLVF | Homsap TRBV2*01 F | Homsap TRBJ1-4*01 F | Homsap TRBD1*01 F | CASSDYNAEKLFF |
|  | Homsap TRAV17*01 F | Homsap TRAJ47*01 F | CATDEEEYGNKLVF | Homsap TRBV27*01 F | Homsap TRBJ1-1*01 F | Homsap TRBD1*01 F | CASSLSPAGSDSEAFF |
|  | Homsap TRAV17*01 F | Homsap TRAJ47*01 F | CATDEEEYGNKLVF | Homsap TRBV20-1*05 (F) | Homsap TRBJ1-6*01 F | Homsap TRBD2*01 F | CSASHPGDSPLHF |
|  | Homsap TRAV4*01 F | Homsap TRAJ23*01 F | CLVEKEEGGKLIF | Homsap TRBV7-9*03 F | Homsap TRBJ2-1*01 F | Homsap TRBD2*01 F | CASSPLDYYNEQFF |
|  | Homsap TRAV38-1*03 (F) | Homsap TRAJ57*01 F | CAFIVPQGGSEKLVF | Homsap TRBV4-1*01 F | Homsap TRBJ1-1*01 F | Homsap TRBD2*01 F | CASSPDPWGVDTEAFF |
|  | Homsap TRAV12-1*01 F | Homsap TRAJ12*01 F | CVVLMDSSYKLIF | Homsap TRBV13*01 F | Homsap TRBJ2-7*02 ORF | Homsap TRBD1*01 F | CASSSLTGGDEQYV |
|  | Homsap TRAV38-1*03 (F) | Homsap TRAJ57*01 F | CAFIVPQGGSEKLVF | Homsap TRBV27*01 F | Homsap TRBJ1-6*01 F | Homsap TRBD1*01 F | CASSLSRAGGDSPLHF |
|  | Homsap TRAV38-1*03 (F) | Homsap TRAJ57*01 F | CAFIVPQGGSEKLVF | Homsap TRBV24-1*01 F | Homsap TRBJ2-1*01 F | Homsap TRBD2*01 F | CATSDFTSPLSYNEQFF |
|  | Homsap TRAV17*01 F | Homsap TRAJ27*01 F | CATDEDTNAGKSTF | Homsap TRBV2*01 F | Homsap TRBJ1-4*01 F | Homsap TRBD1*01 F | CASSDYNAEKLFF |
|  | Homsap TRAV17*01 F | Homsap TRAJ47*01 F | CATDEEEYGNKLVF | Homsap TRBV27*01 F | Homsap TRBJ1-1*01 F | Homsap TRBD1*01 F | CASSLSPAGSDSEAFF |
|  | Homsap TRAV38-2/DV8*01 F | Homsap TRAJ33*01 F | CASVDSNYQLIW | Homsap TRBV27*01 F | Homsap TRBJ1-1*01 F | Homsap TRBD1*01 F | CASSLSPAGSDSEAFF |
|  | Homsap TRAV38-2/DV8*01 F | Homsap TRAJ42*01 F | CAYSYGGSQGNLIF | Homsap TRBV2*01 F | Homsap TRBJ1-4*01 F | Homsap TRBD1*01 F | CASSDYNAEKLFF |
|  | Homsap TRAV12-1*01 F | Homsap TRAJ12*01 F | CVVLMDSSYKLIF | Homsap TRBV20-1*05 (F) | Homsap TRBJ2-3*01 F | Homsap TRBD2*01 F | CSAPGLAGSTDTQYF |
|  | Homsap TRAV17*01 F | Homsap TRAJ47*01 F | CATDEEEYGNKLVF | Homsap TRBV2*01 F | Homsap TRBJ1-4*01 F | Homsap TRBD1*01 F | CASSDYNAEKLFF |
|  | Homsap TRAV17*01 F | Homsap TRAJ47*01 F | CATDEEEYGNKLVF | Homsap TRBV27*01 F | Homsap TRBJ1-1*01 F | Homsap TRBD1*01 F | CASRLSPAGSDSEAFF |
|  | Homsap TRAV17*01 F | Homsap TRAJ10*01 F | CATDAGLTGGGNKLTF | Homsap TRBV27*01 F | Homsap TRBJ1-1*01 F | Homsap TRBD1*01 F | CASSLSPAGSDSEAFF |
|  | Homsap TRAV38-2/DV8*01 F | Homsap TRAJ42*01 F | CAYSYGGSQGNLIF | Homsap TRBV4-1*01 F | Homsap TRBJ1-1*01 F | Homsap TRBD2*01 F | CASSPDPWGVDTEAFF |
|  | Homsap TRAV12-2*01 F | Homsap TRAJ49*01 F | CAVYTGNQFYF | Homsap TRBV2*01 F | Homsap TRBJ1-4*01 F | Homsap TRBD1*01 F | CASSDYNAEKLFF |
|  | Homsap TRAV4*01 F | Homsap TRAJ34*01 F | CLVGDIDTDKLIF | Homsap TRBV2*01 F | Homsap TRBJ1-4*01 F | Homsap TRBD1*01 F | CASSDYNAEKLFF |
|  | Homsap TRAV38-2/DV8*01 F | Homsap TRAJ42*01 F | CAYSYGGSQGNLIF | Homsap TRBV11-1*01 F | Homsap TRBJ2-3*01 F | Homsap TRBD2*01 F | CASSPGPGTIDTDTQYF |
|  | Homsap TRAV17*01 F | Homsap TRAJ47*01 F | CATDEEEYGNKLVF | Homsap TRBV13*01 F | Homsap TRBJ2-7*02 ORF | Homsap TRBD1*01 F | CASSSLTGGDEQYV |
|  | Homsap TRAV17*01 F | Homsap TRAJ47*01 F | CATDEEEYGNKLVF | Homsap TRBV2*01 F | Homsap TRBJ1-4*01 F | Homsap TRBD1*01 F | CASSDYNAEKLFF |
|  |  |  |  | Homsap TRBV27*01 F | Homsap TRBJ1-1*01 F | Homsap TRBD1*01 F | CASSLSPAGSDSEAFF |
|  |  |  |  | Homsap TRBV2*01 F | Homsap TRBJ1-4*01 F | Homsap TRBD1*01 F | CASSDYNAEKLFF |
|  |  |  |  | Homsap TRBV3-1*01 F | Homsap TRBJ2-1*01 F | Homsap TRBD2*01 F | CASSQELALFNEQFF |
|  |  |  |  | Homsap TRBV27*01 F | Homsap TRBJ1-1*01 F | Homsap TRBD1*01 F | CASSLSPAGSDSEAFF |
|  |  |  |  | Homsap TRBV13*01 F | Homsap TRBJ2-7*02 ORF | Homsap TRBD1*01 F | CASSSLTGGDEQYV |
|  |  |  |  | Homsap TRBV27*01 F | Homsap TRBJ1-1*01 F | Homsap TRBD1*01 F | CASSLSPAGSDSEAFF |
|  |  |  |  | Homsap TRBV13*01 F | Homsap TRBJ2-7*02 ORF | Homsap TRBD1*01 F | CASSSLTGGDEQYV |
|  |  |  |  | Homsap TRBV2*01 F | Homsap TRBJ1-4*01 F | Homsap TRBD1*01 F | CASSDYNAEKLFF |
|  |  |  |  | Homsap TRBV2*01 F | Homsap TRBJ1-4*01 F | Homsap TRBD1*01 F | CASSDYNAEKLFF |
|  |  |  |  | Homsap TRBV13*01 F | Homsap TRBJ2-7*02 ORF | Homsap TRBD1*01 F | CASSSLTGGDEQYV |
|  |  |  |  | Homsap TRBV4-1*01 F | Homsap TRBJ1-1*01 F | Homsap TRBD2*01 F | CASSPDPWGVDTEAFF |
|  |  |  |  | Homsap TRBV4-1*01 F | Homsap TRBJ1-1*01 F | Homsap TRBD2*01 F | CASSPDPWGVDTEAFF |
| SARS-CoV | Homsap TRAV17*01 F | Homsap TRAJ27*01 F | CATGAVNTNAGKSTF | Homsap TRBV6-6*01 F | Homsap TRBJ2-5*01 F | Homsap TRBD2*01 F | CASRLTPSGGGQETQYF |
|  | Homsap TRAV19*01 F | Homsap TRAJ13*02 F | CALRRGGYQKVTF | Homsap TRBV4-1*01 F | Homsap TRBJ2-1*01 F | Homsap TRBD1*01 F | CASSQEFVGQLNEQFF |
|  | Homsap TRAV19*01 F | Homsap TRAJ17*01 F | CALHRTPWAAGNKLTF | Homsap TRBV12-4*01 F, or Homsap TRBV12-4*02 (F) | Homsap TRBJ2-2*01 F | Homsap TRBD2*01 F | CASSPLSAGELFF |
|  | Homsap TRAV19*01 F | Homsap TRAJ23*01 F | CALSEGTYNQGGKLIF | Homsap TRBV4-1*01 F | Homsap TRBJ2-7*01 F | Homsap TRBD2*02 F | CASSQDFAGGPYEQYF |
|  | Homsap TRAV23/DV6*01 F | Homsap TRAJ21*01 F | CAASRKDNFNKFYF | Homsap TRBV5-1*01 F | Homsap TRBJ1-2*01 F | Homsap TRBD2*02 F | CASSSGIGLYGYTF |
|  | Homsap TRAV1-2*01 F | Homsap TRAJ9*01 F | CAAHTGGFKTIF | Homsap TRBV27*01 F | Homsap TRBJ2-5*01 F | Homsap TRBD1*01 F | CASSPWEGETQYF |
|  | Homsap TRAV23/DV6*01 F | Homsap TRAJ34*01 F | CAASNRASYNTDKLIF | Homsap TRBV24-1*01 F | Homsap TRBJ2-3*01 F | Homsap TRBD2*01 F | CATRAPPSKGSTDTQYF |
|  | Homsap TRAV27*01 F | Homsap TRAJ49*01 F | CAVTGNQFYF | Homsap TRBV10-3*02 F | Homsap TRBJ1-2*01 F | Homsap TRBD1*01 F | CAIRRGGDGYTF |
|  | Homsap TRAV13-1*01 F | Homsap TRAJ26*01 F | CAASYYGQNFVF | Homsap TRBV7-2*02 F, or Homsap TRBV7-2*03 F | Homsap TRBJ2-3*01 F | Homsap TRBD1*01 F | CASSSPPGQGADTQYF |
|  | Homsap TRAV20*02 F | Homsap TRAJ29*01 F | CAVKSGNTPLVF | Homsap TRBV18*01 F | Homsap TRBJ2-3*01 F | Homsap TRBD1*01 F | CASSRPLDTQYF |
|  | Homsap TRAV2*01 F | Homsap TRAJ30*01 F | CAVGAVLDDKIIF | Homsap TRBV4-1*01 F | Homsap TRBJ2-7*02 ORF | Homsap TRBD1*01 F | CASSQVPTGTEQYV |
|  | Homsap TRAV13-1*01 F | Homsap TRAJ8*01 F | CAGGGFQKLVF | Homsap TRBV9*01 F | Homsap TRBJ2-7*01 F | Homsap TRBD2*02 F | CASSHPTSGRRDYEQYF |
|  | Homsap TRAV29/DV5*01 F | Homsap TRAJ41*01 F | CAASTRDSGYALNF | Homsap TRBV11-2*01 F | Homsap TRBJ1-3*01 F | Homsap TRBD1*01 F | CASSLPQGAGNTIYF |
|  | Homsap TRAV24*01 F | Homsap TRAJ48*01 F | CASYNFGNEKLTF | Homsap TRBV14*01 F | Homsap TRBJ2-1*01 F | Homsap TRBD2*01 F | CASSQPTSGGSAEQFF |
|  | Homsap TRAV3*01 F | Homsap TRAJ13*02 F | CAVREGSGGYQKVTF | Homsap TRBV5-1*01 F | Homsap TRBJ2-1*01 F | Homsap TRBD2*02 F | CASSFAGPYNEQFF |
|  | Homsap TRAV13-1*01 F | Homsap TRAJ40*01 F | CAAIDGTSGTYKYIF | Homsap TRBV5-6*01 F | Homsap TRBJ2-4*01 F | Homsap TRBD1*01 F | CASSGTGSKNIQYF |
|  | Homsap TRAV4*01 F | Homsap TRAJ37*01 F | CLVGSSGNTGKLIF | Homsap TRBV9*01 F | Homsap TRBJ2-4*01 F | Homsap TRBD2*01 F | CASSASGLAGAKNIQYF |
|  | Homsap TRAV9-2*01 F | Homsap TRAJ52*01 F | CALTNAGGTSYGKLTF | Homsap TRBV5-5*02 (F) | Homsap TRBJ1-5*01 F | Homsap TRBD1*01 F | CASSLIHREDQPQHF |
|  | Homsap TRAV1-2*01 F | Homsap TRAJ11*01 F | CAANSGYSTLTF | Homsap TRBV7-6*01 F | Homsap TRBJ2-7*01 F | Homsap TRBD1*01 F | CASSLLLDSSYEQYF |
|  | Homsap TRAV38-2/DV8*01 F | Homsap TRAJ12*01 F | CAQEDSSYKLIF | Homsap TRBV20-1*05 (F) | Homsap TRBJ1-2*01 F | Homsap TRBD2*01 F | CSAPNLDYGGSSYGYTF |
|  | Homsap TRAV13-2*01 F | Homsap TRAJ41*01 F | CAEKVSNSGYALNF | Homsap TRBV20-1*05 (F) | Homsap TRBJ2-1*01 F | Homsap TRBD2*02 F | CSATTIRGPGNEQFF |
|  | Homsap TRAV17*01 F | Homsap TRAJ40*01 F | CATGSFTSGTYKYIF | Homsap TRBV9*01 F | Homsap TRBJ2-1*01 F | Homsap TRBD2*01 F | CASNPSGSYNEQFF |
|  | Homsap TRAV13-2*01 F | Homsap TRAJ41*01 F | CAERGHSGYALNF | Homsap TRBV2*01 F | Homsap TRBJ1-5*01 F | Homsap TRBD2*01 F | CASSEALRGSVGQPQHF |
|  | Homsap TRAV13-1*01 F | Homsap TRAJ26*01 F | CAASISDYGQNFVF | Homsap TRBV7-3*01 F | Homsap TRBJ2-1*01 F | Homsap TRBD2*01 F | CASSLGGAGYNEQFF |
|  | Homsap TRAV29/DV5*01 F | Homsap TRAJ41*01 F | CAASTRDSGYALNF | Homsap TRBV4-1*01 F | Homsap TRBJ2-5*01 F | Homsap TRBD1*01 F | CASSQDSIWTLVQETQYF |
|  | Homsap TRAV2*01 F | Homsap TRAJ9*01 F | CAVGNTGGFKTIF | Homsap TRBV5-6*01 F | Homsap TRBJ2-7*01 F | Homsap TRBD1*01 F | CASRAGGGGGYEQYF |
|  | Homsap TRAV17*01 F | Homsap TRAJ13*02 F | CAPRAPWGYQKVTF | Homsap TRBV20-1*05 (F) | Homsap TRBJ2-1*01 F | Homsap TRBD2*01 F | CSARDEGGLAYEQFF |
|  | Homsap TRAV38-2/DV8*01 F | Homsap TRAJ43*01 F | CAYKGNDMRF | Homsap TRBV2*01 F | Homsap TRBJ2-2*01 F | Homsap TRBD1*01 F | CATPSEVGNTGELFF |
|  | Homsap TRAV29/DV5*01 F | Homsap TRAJ40*01 F | CAATVSGTYKYIF | Homsap TRBV2*01 F | Homsap TRBJ2-2*01 F |  | CASSAPSNTGELFF |
|  | Homsap TRAV26-1*02 F | Homsap TRAJ49*01 F | CIALNQFYF | Homsap TRBV4-2*01 F | Homsap TRBJ2-1*01 F | Homsap TRBD2*02 F | CASSQGGERVQFF |
|  | Homsap TRAV38-1*03 (F) | Homsap TRAJ47*02 (F) | CAFEGGAYGNKLVF | Homsap TRBV7-9*03 F | Homsap TRBJ2-1*01 F | Homsap TRBD2*02 F | CASSSSLAGGGSSYNEQFF |
|  | Homsap TRAV23/DV6*01 F | Homsap TRAJ24*02 F | CAASMTDSWGKLQF | Homsap TRBV4-1*01 F | Homsap TRBJ2-2*01 F | Homsap TRBD2*01 F | CASSQLPTSSFAGELFF |
|  | Homsap TRAV21*02 (F) | Homsap TRAJ33*01 F | CAVQADSNYQLIW | Homsap TRBV24-1*01 F | Homsap TRBJ2-7*01 F | Homsap TRBD1*01 F | CATSVGNSEQYF |
|  | Homsap TRAV13-1*01 F | Homsap TRAJ27*01 F | CAAPRNTNAGKSTF | Homsap TRBV4-1*01 F | Homsap TRBJ2-1*01 F | Homsap TRBD1*01 F | CASSPNAGGTYNEQFF |
|  | Homsap TRAV36/DV7*04 (F) | Homsap TRAJ49*01 F | CAVDGQFYF | Homsap TRBV5-6*01 F | Homsap TRBJ2-2*01 F | Homsap TRBD1*01 F | CASSLGGYTGELFF |
|  | Homsap TRAV19*01 F | Homsap TRAJ38*01 F | CALPNAGNNRKLIW | Homsap TRBV7-9*03 F | Homsap TRBJ1-5*01 F | Homsap TRBD2*02 F | CASSRGEEPNNQPQHF |
|  | Homsap TRAV19*01 F | Homsap TRAJ20*01 F | CALSGPNDYKLSF | Homsap TRBV12-3*01 F | Homsap TRBJ2-7*02 ORF | Homsap TRBD2*01 F | CASMSRGARREQYV |
|  | Homsap TRAV6*07 F | Homsap TRAJ23*01 F | CALGAYNQGGKLIF | Homsap TRBV6-1*01 F | Homsap TRBJ2-7*02 ORF | Homsap TRBD2*01 F | CASSWDSGYEQYV |
|  | Homsap TRAV35*02 (F) | Homsap TRAJ30*01 F | CAGQTTYDKIIF | Homsap TRBV12-4*01 F, or Homsap TRBV12-4*02 (F) | Homsap TRBJ2-3*01 F | Homsap TRBD2*02 F | CASSPWSGRADTQYF |
|  | Homsap TRAV25*01 F (see comment) | Homsap TRAJ52*01 F | CAGPVLGGTSYGKLTF | Homsap TRBV5-6*01 F | Homsap TRBJ2-7*01 F | Homsap TRBD1*01 F | CASSLWGAGAYEQYF |
|  | Homsap TRAV21*01 F | Homsap TRAJ57*01 F | CAVLGGSEKLVF | Homsap TRBV5-6*01 F | Homsap TRBJ2-7*01 F | Homsap TRBD1*01 F | CASSLWGAGAYEQYF |
|  | Homsap TRAV20*02 F | Homsap TRAJ24*02 F | CATTDSWGKLQF | Homsap TRBV27*01 F | Homsap TRBJ2-3*01 F | Homsap TRBD1*01 F | CASKDRGPGDTQYF |
|  | Homsap TRAV23/DV6*01 F | Homsap TRAJ10*01 F | CAASEILTGGGNKLTF | Homsap TRBV27*01 F | Homsap TRBJ2-2*01 F | Homsap TRBD1*01 F | CASKGSNTGELFF |
|  | Homsap TRAV29/DV5*01 F | Homsap TRAJ30*01 F | CAASPLNRDDKIIF | Homsap TRBV20-1*05 (F) | Homsap TRBJ1-4*01 F | Homsap TRBD1*01 F | CSARNYGTANEKLFF |
|  | Homsap TRAV21*01 F | Homsap TRAJ41*01 F | CAVEDSGYALNF | Homsap TRBV12-3*01 F | Homsap TRBJ2-1*01 F | Homsap TRBD1*01 F | CASSLDPGGFNSNEQFF |
|  | Homsap TRAV8-3*02 (F) | Homsap TRAJ44*01 F | CAVGGKTGTASKLTF | Homsap TRBV29-1*01 F | Homsap TRBJ2-1*01 F | Homsap TRBD2*01 F | CSVEDHGLVDEQFF |
|  | Homsap TRDV1*01 F | Homsap TRAJ40*01 F | CALGELSGSGTYKYIF | Homsap TRBV13*01 F | Homsap TRBJ1-1*01 F | Homsap TRBD1*01 F | CASSSQGARDTEAFF |
|  | Homsap TRAV19*01 F | Homsap TRAJ47*01 F | CALSEAKYGNKLVF | Homsap TRBV4-1*01 F | Homsap TRBJ2-7*01 F | Homsap TRBD2*02 F | CASSRAGGTYEQYF |
|  | Homsap TRAV26-1*01 F | Homsap TRAJ13*02 F | CIVRERGYQKVTF | Homsap TRBV9*01 F | Homsap TRBJ2-3*01 F |  | CASSVSDTQYF |
|  | Homsap TRAV5*01 F | Homsap TRAJ53*01 F | CAEWGSNYKLTF | Homsap TRBV19*01 F | Homsap TRBJ2-1*01 F | Homsap TRBD2*02 F | CASSITSGTNEQFF |
|  | Homsap TRAV3*01 F | Homsap TRAJ28*01 F | CAVLDPGAGSYQLTF | Homsap TRBV6-6*01 F, or Homsap TRBV6-6*04 (F) (see comment) | Homsap TRBJ2-7*01 F | Homsap TRBD2*02 F | CASSTQGSGSWVEQYF |
|  | Homsap TRAV29/DV5*01 F | Homsap TRAJ41*01 F | CAASSGYALNF | Homsap TRBV20-1*05 (F) | Homsap TRBJ1-1*01 F | Homsap TRBD2*01 F | CSAVTVNTEAFF |
|  | Homsap TRAV35*02 (F) | Homsap TRAJ47*02 (F) | CAGMEYGNKLVF | Homsap TRBV5-5*02 (F) | Homsap TRBJ2-7*02 ORF | Homsap TRBD2*01 F | CASSWTSGYEQYV |
|  | Homsap TRAV1-2*01 F | Homsap TRAJ33*01 F | CAVRDLDSNYQLIW | Homsap TRBV9*01 F | Homsap TRBJ2-7*01 F | Homsap TRBD2*02 F | CASSVGAGTFTYEQYF |
|  | Homsap TRAV20*02 F | Homsap TRAJ48*01 F | CAPNFGNEKLTF | Homsap TRBV4-1*01 F | Homsap TRBJ1-1*01 F | Homsap TRBD2*02 F | CASSQERGMMNTEAFF |
|  | Homsap TRAV38-2/DV8*01 F | Homsap TRAJ12*01 F | CAQEDSSYKLIF | Homsap TRBV7-2*02 F, or Homsap TRBV7-2*03 F | Homsap TRBJ2-3*01 F | Homsap TRBD1*01 F | CASSLAGGQGTDTQYF |
|  | Homsap TRAV39*01 F | Homsap TRAJ43*01 F | CAVDTYNNNDMRF | Homsap TRBV21-1*01 P | Homsap TRBJ2-7*02 ORF | Homsap TRBD1*01 F | CASRDRQRDGQYV |
|  | Homsap TRAV38-1*01 F | Homsap TRAJ42*01 F | CAINYGGSQGNLIF | Homsap TRBV14*01 F | Homsap TRBJ1-1*01 F | Homsap TRBD1*01 F | CASSQGRTAEAFF |
|  |  |  |  | Homsap TRBV5-1*01 F | Homsap TRBJ2-2*01 F | Homsap TRBD1*01 F | CASSSPDSTTGELFF |
|  |  |  |  | Homsap TRBV5-6*01 F | Homsap TRBJ1-4*01 F | Homsap TRBD1*01 F | CASSLGGAYEKLFF |
|  |  |  |  | Homsap TRBV5-8*01 F | Homsap TRBJ2-1*01 F | Homsap TRBD2*02 F | CASSFRETYNEQFF |
|  |  |  |  | Homsap TRBV27*01 F | Homsap TRBJ2-7*01 F | Homsap TRBD1*01 F | CASSLLGDRVHEQYF |
|  |  |  |  | Homsap TRBV10-3*03 [F] | Homsap TRBJ2-1*01 F | Homsap TRBD2*02 F | CAISETVAGGPGNEQFF |
|  |  |  |  | Homsap TRBV27*01 F | Homsap TRBJ2-3*01 F | Homsap TRBD1*01 F | CASGDIADTQYF |
|  |  |  |  | Homsap TRBV29-1*01 F | Homsap TRBJ2-3*01 F | Homsap TRBD2*01 F | CSVELRADTQYF |
|  |  |  |  | Homsap TRBV4-2*01 F | Homsap TRBJ1-2*01 F | Homsap TRBD1*01 F | CASSQGGDRGLQLGYTF |
|  |  |  |  | Homsap TRBV12-4*01 F, or Homsap TRBV12-4*02 (F) | Homsap TRBJ2-7*01 F | Homsap TRBD2*01 F | CASRTSATYEQYF |
|  |  |  |  | Homsap TRBV4-2*01 F | Homsap TRBJ2-1*01 F | Homsap TRBD2*02 F | CASSQDGLAGNNEQFF |
|  |  |  |  | Homsap TRBV24-1*01 F | Homsap TRBJ2-5*01 F | Homsap TRBD2*01 F | CATSDSSSGPDPQETQYF |
|  |  |  |  | Homsap TRBV28*01 F | Homsap TRBJ2-7*01 F | Homsap TRBD2*01 F | CASSLGRDLLYEQYF |
|  |  |  |  | Homsap TRBV4-1*01 F | Homsap TRBJ1-1*01 F | Homsap TRBD1*01 F | CASSQETGGTEAFF |
|  |  |  |  | Homsap TRBV4-1*01 F | Homsap TRBJ1-4*01 F | Homsap TRBD1*01 F | CASRTRDRGEKLFF |
|  |  |  |  | Homsap TRBV7-3*01 F | Homsap TRBJ2-1*01 F | Homsap TRBD2*01 F | CASSELDASGVYNEQFF |
|  |  |  |  | Homsap TRBV6-5*01 F | Homsap TRBJ2-1*01 F | Homsap TRBD1*01 F | CASSELNRLGYNEQFF |
|  |  |  |  | Homsap TRBV20-1*05 (F) | Homsap TRBJ2-1*01 F | Homsap TRBD2*01 F | CSARDEGGLAYEQFF |
|  |  |  |  | Homsap TRBV5-1*01 F | Homsap TRBJ2-1*01 F | Homsap TRBD1*01 F | CASSSRDVLNEQFF |
|  |  |  |  | Homsap TRBV20-1*05 (F) | Homsap TRBJ2-3*01 F | Homsap TRBD2*01 F | CSYLADTQYF |
|  |  |  |  | Homsap TRBV3-1*01 F | Homsap TRBJ2-5*01 F | Homsap TRBD2*02 F | CASSQDLASGRPREETQYF |
|  |  |  |  | Homsap TRBV5-1*01 F | Homsap TRBJ2-7*01 F | Homsap TRBD1*01 F | CASSSGQGVYEQYF |
|  |  |  |  | Homsap TRBV21-1*01 P | Homsap TRBJ1-1*01 F | Homsap TRBD1*01 F | CASSKALSGTGGHTEAFF |
|  |  |  |  | Homsap TRBV5-1*01 F | Homsap TRBJ2-2*01 F |  | CASSTHGELFF |
|  |  |  |  | Homsap TRBV3-1*01 F | Homsap TRBJ2-3*01 F | Homsap TRBD1*01 F | CASSAVRGSNTDTQYF |
|  |  |  |  | Homsap TRBV24-1*01 F | Homsap TRBJ2-7*02 ORF | Homsap TRBD2*01 F | CATSALRTSGPQNYEQYV |
|  |  |  |  | Homsap TRBV9*01 F | Homsap TRBJ1-2*01 F | Homsap TRBD1*01 F | CASSTHLATGALGYTF |
|  |  |  |  | Homsap TRBV5-1*01 F | Homsap TRBJ2-1*01 F | Homsap TRBD2*02 F | CASSLGSEEFF |
|  |  |  |  | Homsap TRBV6-1*01 F | Homsap TRBJ2-1*01 F | Homsap TRBD2*01 F | CASSEERLAEVIYNEQFF |
|  |  |  |  | Homsap TRBV5-1*01 F | Homsap TRBJ2-1*01 F | Homsap TRBD2*01 F | CASSLGTVEQFF |
|  |  |  |  | Homsap TRBV29-1*01 F | Homsap TRBJ1-3*01 F | Homsap TRBD1*01 F | CSVAAKRGGIGNTIYF |
|  |  |  |  | Homsap TRBV4-1*01 F | Homsap TRBJ2-2*01 F | Homsap TRBD1*01 F | CASSQDSAGRTELFF |
|  |  |  |  | Homsap TRBV13*01 F | Homsap TRBJ2-2*01 F | Homsap TRBD2*01 F | CASSTPGTSNTGELFF |
|  |  |  |  | Homsap TRBV13*01 F | Homsap TRBJ1-1*01 F | Homsap TRBD2*02 F | CASSYTGRRNTEAFF |
|  |  |  |  | Homsap TRBV5-1*01 F | Homsap TRBJ2-7*02 ORF | Homsap TRBD1*01 F | CASSLTGPVGYEQYV |
|  |  |  |  | Homsap TRBV24-1*01 F | Homsap TRBJ2-2*01 F | Homsap TRBD1*01 F | CATSEGSYTGELFF |
|  |  |  |  | Homsap TRBV20-1*05 (F) | Homsap TRBJ1-5*01 F | Homsap TRBD1*01 F | CSAREPGGVENQPQHF |
|  |  |  |  | Homsap TRBV29-1*01 F | Homsap TRBJ1-5*01 F |  | CSANNQPQHF |
|  |  |  |  | Homsap TRBV20-1*05 (F) | Homsap TRBJ2-1*01 F | Homsap TRBD2*02 F | CSARFAGVSYNEQFF |
|  |  |  |  | Homsap TRBV9*01 F | Homsap TRBJ2-7*01 F | Homsap TRBD2*02 F | CANSPSGSFDEQYF |
|  |  |  |  | Homsap TRBV12-3*01 F | Homsap TRBJ2-1*01 F | Homsap TRBD2*01 F | CASGNRLYNEQFF |
|  |  |  |  | Homsap TRBV9*01 F | Homsap TRBJ2-3*01 F | Homsap TRBD1*01 F | CASSLGTLSTQYF |
|  |  |  |  | Homsap TRBV5-1*01 F | Homsap TRBJ2-3*01 F | Homsap TRBD2*02 F | CASSGSEEGDTQNF |
|  |  |  |  | Homsap TRBV19*01 F | Homsap TRBJ1-1*01 F | Homsap TRBD2*02 F | CASTQRGSTEAFF |
|  |  |  |  | Homsap TRBV30*01 F | Homsap TRBJ2-3*01 F | Homsap TRBD1*01 F | CAWRAASSTDTQYF |
|  |  |  |  | Homsap TRBV20-1*05 (F) | Homsap TRBJ2-1*01 F | Homsap TRBD2*01 F | CSARDEGGLAYEQFF |
|  |  |  |  | Homsap TRBV20-1*05 (F) | Homsap TRBJ2-1*01 F | Homsap TRBD2*01 F | CSARDEGGLAYEQFF |
|  |  |  |  | Homsap TRBV20-1*05 (F) | Homsap TRBJ2-1*01 F | Homsap TRBD2*02 F | CSAITAGGPMNEQFF |

**Table S4.**

**The characteristics of participants.**

|  | **Macheng** | | **Shijiazhuang** | **HC** |
| --- | --- | --- | --- | --- |
|  | **6m (n=76)** | **12m (n=74)** | **(n=45)** | **(n=28)** |
| **Demographic information** |  |  |  |  |
| Sex, male/female | 38/38 | 37/37 | 12/33 | 15/13 |
| Age, median(IQR) | 49(41,57) | 49(41,56) | 42(29,59) | 38(26,61) |
|  |  |  |  |  |
| **Clinical phenotype** |  |  |  |  |
| Asymptomatic,(%) | 8/76(11%) | 6/74(8%) | 8/45(18%) |  |
| Mild,(%) | 36/76(47%) | 36/74(49%) | 5/45(11%) |  |
| Moderate,(%) | 23/76(30%) | 26/74(35%) | 32/45(71%) |  |
| Severe,(%) | 9/76(12%) | 6/74(8%) | 0/45(0%) |  |
|  |  |  |  |  |
| **Follow-up information** |  |  |  |  |
| Days after onset,median(IQR) | 178(172,181) | 352(346,355) | 200(196,202) |  |

**Table S5.**

**The two and three-dimensional peptide matrix pools.**

1. **Spike protein: 199 peptides in total 30 pools including 10 pools in 1^st^ dimension, 10 pools in 2^nd^ dimension and 10 pools in 3^rd^ dimension^a^**

|  | **Pool-1** | | **Pool-2** | | **Pool-3** | | **Pool-4** | | **Pool-5** | |  |
| --- | --- | --- | --- | --- | --- | --- | --- | --- | --- | --- | --- |
| **Pool-11** | S1 | S2 | S3 | S4 | S5 | S6 | S7 | S8 | S9 | S10 | **Pool-21** |
|  | S11 | S12 | S13 | S14 | S15 | S16 | S17 | S18 | S19 | S20 |  |
| **Pool-12** | S21 | S22 | S23 | S24 | S25 | S26 | S27 | S28 | S29 | S30 | **Pool-22** |
|  | S31 | S32 | S33 | S34 | S35 | S36 | S37 | S38 | S39 | S40 |  |
| **Pool-13** | S41 | S42 | S43 | S44 | S45 | S46 | S47 | S48 | S49 | S50 | **Pool-23** |
|  | S51 | S52 | S53 | S54 | S55 | S56 | S57 | S58 | S59 | S60 |  |
| **Pool-14** | S61 | S62 | S63 | S64 | S65 | S66 | S67 | S68 | S69 | S70 | **Pool-24** |
|  | S71 | S72 | S73 | S74 | S75 | S76 | S77 | S78 | S79 | S80 |  |
| **Pool-15** | S81 | S82 | S83 | S84 | S85 | S86 | S87 | S88 | S89 | S90 | **Pool-25** |
|  | S91 | S92 | S93 | S94 | S95 | S96 | S97 | S98 | S99 | S100 |  |
|  |  | |  | |  | |  | |  | |  |
|  | **Pool-6** | | **Pool-7** | | **Pool-8** | | **Pool-9** | | **Pool-10** | |  |
| **Pool-16** | S101 | S102 | S103 | S104 | S105 | S106 | S107 | S108 | S109 | S110 | **Pool-26** |
|  | S111 | S112 | S113 | S114 | S115 | S116 | S117 | S118 | S119 | S120 |  |
| **Pool-17** | S121 | S122 | S123 | S124 | S125 | S126 | S127 | S128 | S129 | S130 | **Pool-27** |
|  | S131 | S132 | S133 | S134 | S135 | S136 | S137 | S138 | S139 | S140 |  |
| **Pool-18** | S141 | S142 | S143 | S144 | S145 | S146 | S147 | S148 | S149 | S150 | **Pool-28** |
|  | S151 | S152 | S153 | S154 | S155 | S156 | S157 | S158 | S159 | S160 |  |
| **Pool-19** | S161 | S162 | S163 | S164 | S165 | S166 | S167 | S168 | S169 | S170 | **Pool-29** |
|  | S171 | S172 | S173 | S174 | S175 | S176 | S177 | S178 | S179 | S180 |  |
| **Pool-20** | S181 | S182 | S183 | S184 | S185 | S186 | S187 | S188 | S189 | S190 | **Pool-30** |
|  | S191 | S192 | S193 | S194 | S195 | S196 | S197 | S198 | S199 |  |  |

1. In the tables, Pool-1 to 10 contained corresponding two columns of peptides, Pool-11 to 20 contained corresponding two rows of peptides, and Pool-21 to 30 contained the peptides of the same color.
2. **Membrane and nucleocapsid protein: 86 peptides in total 19 pools including 10 pools in 1^st^ dimension and 9 pools in 2^nd^ dimension**

|  | **Pool-31** | **Pool-32** | **Pool-33** | **Pool-34** | **Pool-35** | **Pool-36** | **Pool-37** | **Pool-38** | **Pool-39** | **Pool-40** |
| --- | --- | --- | --- | --- | --- | --- | --- | --- | --- | --- |
| **Pool-41** | M1 | M2 | M3 | M4 | M5 | M6 | M7 | M8 | M9 | M10 |
| **Pool-42** | M11 | M12 | M13 | M14 | M15 | M16 | M17 | M18 | M19 | M20 |
| **Pool-43** | M21 | M22 | M23 | M24 | M25 | M26 | M27 | M28 | M29 | N1 |
| **Pool-44** | N2 | N3 | N4 | N5 | N6 | N7 | N8 | N9 | N10 | N11 |
| **Pool-45** | N12 | N13 | N14 | N15 | N16 | N17 | N18 | N19 | N20 | N21 |
| **Pool-46** | N22 | N23 | N24 | N25 | N26 | N27 | N28 | N29 | N30 | N31 |
| **Pool-47** | N32 | N33 | N34 | N35 | N36 | N37 | N38 | N39 | N40 | N41 |
| **Pool-48** | N42 | N43 | N44 | N45 | N46 | N47 | N48 | N49 | N50 | N51 |
| **Pool-49** | N52 | N53 | N54 | N55 | N56 | N57 |  |  |  |  |
